# Cross-species outlier detection reveals different evolutionary pressures between sister species

**DOI:** 10.1111/nph.12896

**Published:** 2014-06-18

**Authors:** Catherine I Cullingham, Janice E K Cooke, David W Coltman

**Affiliations:** Department of Biological Sciences, University of AlbertaBiological Sciences Building, Edmonton, AB, T6G 2E9, Canada

**Keywords:** adaptive variation, environmental correlation, jack pine (*Pinus banksiana*), lodgepole pine (*Pinus contorta* var. *latifolia*), outlier detection, selection

## Abstract

- Lodgepole pine (*Pinus contorta* var. *latifolia*) and jack pine (*Pinus banksiana*) hybridize in western Canada, an area of recent mountain pine beetle range expansion. Given the heterogeneity of the environment, and indications of local adaptation, there are many unknowns regarding the response of these forests to future outbreaks. To better understand this we aim to identify genetic regions that have adaptive potential.
- We used data collected on 472 single nucleotide polymorphism (SNP) loci from 576 tree samples collected across 13 lodgepole pine-dominated sites and four jack pine-dominated sites. We looked at the relationship of genetic diversity with the environment, and we identified candidate loci using both frequency-based (arlequin and bayescan) and correlation-based (matsam and bayenv) methods.
- We found contrasting relationships between environmental variation and genetic diversity for the species. While we identified a number of candidate outliers (34 in lodgepole pine, 25 in jack pine, and 43 interspecific loci), we did not find any loci in common between lodgepole and jack pine. Many of the outlier loci identified were correlated with environmental variation.
- Using rigorous criteria we have been able to identify potential outlier SNPs. We have also found evidence of contrasting environmental adaptations between lodgepole and jack pine which could have implications for beetle spread risk.

## Introduction

A fundamental goal of evolutionary biology is to identify adaptive variation within a genome. While in the past this may have been hampered by a paucity of appropriate methods and limited access to relevant genomic data, this area is rapidly expanding. With the advent of single nucleotide polymorphism (SNP)-chip genotyping and next-generation sequencing (Hudson, 2008), and an increase in bioinformatic capability, we are better able to study nonmodel organisms and begin to identify and characterize adaptive variation (Luikart *et al.*, 2003; Ekblom & Galindo, 2011). This comes at a critical time where many species are faced with changing environments as a result of anthropogenic stresses, invasive species and climate change (Thomas *et al.*, 2004; Hoffman & Sgrò, 2011).

Forest trees present an interesting system in which to study adaptation as many are sessile and long-lived, with large out-breeding populations. This combination of characteristics could potentially result in maladapted forests if there is insufficient time for them to respond at the genome level to rapid environmental change (Aitken *et al.*, 2008; Lindner *et al.*, 2010). For instance, climate change may lead to rapidly altered forest–pest interactions such as more severe pest outbreaks and changing insect distributions (Logan *et al.*, 2003; Lovett *et al.*, 2006; Bentz *et al.*, 2010). However, resiliency may be increased through introgressive hybridization exhibited by many forest species, as it can result in enhanced ecological and physiological tolerance and adaptive potential (Lewontin & Birch, 1966; Grant & Grant, 2008; Siol *et al.*, 2010). Through understanding sources of adaptive variation we can promote healthy forests through improved seed-stock selection and assisted migration (McLachlan *et al.*, 2007; Aitken *et al.*, 2008).

Jack pine (*Pinus banksiana*) is a keystone species of the boreal forest and has recently been identified as a new host for mountain pine beetle (Cullingham *et al.*, 2011). The host and range shift of the beetle has resulted from an unprecedented outbreak originating in lodgepole pine (*Pinus contorta* var. *latifolia*) host populations, a sister species to jack pine (Wheeler *et al.*, 1983; Parks *et al.*, 2012). These species hybridize in Alberta and the Northwest Territories, which has allowed for gene introgression in both directions (Cullingham *et al.*, 2013). These species occur across heterogeneous landscapes and demonstrate heritable adaptive variation across environmental gradients (Carlson *et al.*, 1999; Rehfeldt *et al.*, 1999; Yeaman & Jarvis, 2006; Rweyongeza *et al.*, 2007) suggesting local adaptation. Tree condition plays an important role in determining susceptibility to attack (Larsson, *et al*. 1993; Christiansen, *et al*. 1987). Tree condition is determined in part by the environment and the tree's ability to respond to changing conditions. Therefore, environmental adaptations may play an important role in understanding stand susceptibility. There is also potential for adaptation to mountain pine beetle in lodgepole pine, evidenced by greater mountain pine beetle reproduction in naïve stands compared with historically exposed stands (Cudmore *et al.*, 2010). Identifying genes that are potentially adaptive in lodgepole and jack pine will allow us to begin to understand how mountain pine beetle may impact naïve lodgepole pine, and a new host species.

To explore the adaptive potential of lodgepole and jack pine, we investigated both intra- and interspecific variation. We expected to find candidates of adaptive variation within both of these species that were not shared, as these species have been exposed to different evolutionary pressures and their distributions are well defined by dissimilar environments (Cullingham *et al.*, 2012). Identifying candidates using interspecific comparison is a relatively recent approach (e.g. Hamilton *et al.*, 2013). From this comparison we expected to identify loci that are highly conserved (negative selection) between the species as a result of their physiological or developmental importance (Fulton *et al.*, 2002), and candidates that are divergent (positive selection) as a result of separate evolutionary histories.

Simulation and pilot studies advise the use of multiple methods to identify candidates of adaptive variation (Narum & Hess, 2011; Nunes *et al.*, 2011; Gosset & Bierne, 2012; De Mita *et al.*, 2013). Therefore, we applied two widely used approaches to detect adaptive variation using genotypic data. The most widely used, the outlier approach, is based on the assumption that loci under selection will have greater or lower variance than neutral loci, suggesting either divergent or stabilizing selection, respectively (Lewontin & Krakauer, 1973). This outlier concept is implemented in a number of different packages (e.g. Beaumont & Nichols, 1996; Balding, 2003; Beaumont & Balding, 2004; Excoffier *et al.*, 2009). An alternative to the outlier approach is to identify spatial correlations of allele frequencies with environmental variables (Endler, 1986). The idea is that environmental correlations will occur when the allele frequency at a locus is influenced by the environment and is therefore under environmental selection. This approach, while more sensitive to finding putative adaptive loci (Hancock *et al.*, 2010; De Mita *et al.*, 2013), is limited to identifying loci that are correlated with environmental variables assumed to be of importance.

To complete these analyses we generated SNP profiles for 546 individuals of lodgepole pine, jack pine and their hybrids across a portion of their range. These SNP resources were developed from 454 transcriptome data for each species exposed to drought and mechanical wounding to mimic mountain pine beetle attack (Cullingham *et al.*, 2013). These data were separated into three subsets for analysis: pure lodgepole pine (13 sites), pure jack pine (four sites) and the two species combined. We identified outlier candidates using arlequin (Excoffier & Lischer, 2010) and bayescan (Foll & Gaggiotti, 2008), and environmental correlations using matsam (Joost *et al.*, 2008) and bayenv (Coop *et al.*, 2010). As we are looking at the importance of adaptive polymorphisms to understand tree response to mountain pine beetle spread risk, we will consider ‘true’ positives to be loci that have adaptive polymorphisms that appear to be under selection. Hybrid individuals were used to estimate genomic clines to identify loci that introgress significantly differently from neutral expectations using introgress (Gompert & Buerkle, 2010). This study is an important step towards identifying and understanding the genes underlying adaptive genetic variation within and between these hybridizing, sister species that have intersecting distributions, yet distinct habitat preferences.

## Materials and Methods

### SNP data

To identify outlier loci within and between species, we used samples of lodgepole (*Pinus contorta* Dougl. ex Loud. var. *latifolia*), jack pine (*Pinus banksiana*, Lamb) and their interspecific hybrids that have been previously characterized at SNP loci described in Cullingham *et al*. (2013). Briefly, 546 samples across 17 locations (Table 1) were characterized at 472 loci: 39 of these were exclusive to lodgepole pine (37 were monomorphic in jack pine, and two did not amplify), 34 were exclusive to jack pine (monomorphic in lodgepole pine), and the remaining 399 were common across species. The data were divided into three subsets for subsequent analyses, except where noted. The first and second subsets consisted of pure lodgepole pine and jack pine (as estimated using genotypic information in Cullingham *et al.*, 2013) considered separately with individuals grouped by sampling site. We used the level of sample site because different sample sites reflect different environmental conditions, and these species exhibit heritable adaptive variation across environmental gradients despite the relative absence of large-scale population structure (Yang *et al.*, 1996; Saenz-Romero *et al.*, 2001; Cullingham *et al.*, 2011, 2013). The third subset comprised all pure lodgepole and jack pine to identify potential adaptive variation across the species barrier, and to identify loci potentially under balancing selection; this subset is hereafter referred to as the ‘combined subset’.

**Table 1:** Location and sample size for the lodgepole pine (*Pinus contorta* var. *latifolia*), jack pine (*Pinus banksiana*), and their interspecific hybrid sampling sites; the breakdown of species distribution at each of the sites is based on clustering analysis using structure (Pritchard *et al.*, 2000) completed in Cullingham *et al*. (2013)

Sample site	*N*	Latitude	Longitude	Lodgepole pine	Jack pine	Hybrids
Canmore	31	50.85930	−115.34727	31		
Conklin	33	55.63323	−111.07812		30	3
Crowsnest Pass	33	49.62875	−114.69402	33		
Cypress Hills	31	49.59338	−110.03555	31		
Fairview	34	56.12779	−118.54754	21		13
Fox Creek	32	54.70253	−117.02108	28		4
Ft. McMurray	34	56.20358	−111.68987		19	15
Golden	35	51.32091	−116.80177	35		
Grande Prairie	32	55.08760	−118.75566	13		19
Kootenay/Yoho	33	50.91233	−116.03792	33		
Ontario	25	48.92822	−92.08884		25	
Saskatchewan	26	53.55246	−106.47227		26	
Sparwood	33	49.81557	−114.87127	33		
Tumbler Ridge	33	54.91308	−121.23003	33		
Valemount	34	52.88225	−119.30085	34		
Wabasca	34	55.49922	−114.04370	10		24
Willmore-Kakwa	33	53.71293	−119.74398	33		

Observed and expected heterozygosity, and the fixation index for each SNP within the lodgepole and jack pine subsets were estimated separately using genalex ver. 6.5 (Peakall & Smouse, 2012). We estimated linkage using genepop ver. 4.2 (Rousset, 2008) and used *q*-value estimates calculated in the R (R Development Core Team, 2011) package qvalue (Dabney *et al.*, 2013) to determine the number of significant comparisons based on the false discovery rate (Storey, 2002).

Annotation of all successfully genotyped SNPs was completed using the compilation of results from multiple database searches of the full contigs using tblastx (Altschul *et al.*, 1990): TAIR9 (Lamesch *et al.*, 2012), NCBI – nonredundant (NR) protein database filtered for plant taxa and the Arborea white spruce (*Picea glauca*) gene catalogue (Rigault *et al.*, 2011). We used only those matches with *E*-values < 10^−8^ to limit matches representing small fragments of high similarity. Sequences were also annotated with gene ontology (GO) terms using blast2go (Conesa *et al.*, 2005) when available.

### Environmental data

To test the effects of environment on genetic diversity we required environmental data at sampling locations. Using the geographical coordinates and elevation estimated by GPS where trees were sampled, we obtained environmental data previously shown to be important for both habitat specialization (Sykes, 2001; Cullingham *et al.*, 2012) and mountain pine beetle defence response (Lusebrink *et al.*, 2011; Arango-Velez *et al.*, 2014). We obtained data for the following 10 variables from the Canadian Forest Service (CFS) website (http://cfs.nrcan.gc.ca/projects/3) calculated using a 30-yr average (1931–1960) from weather stations interpolated for Canada using ANNUSPLIN (Hutchinson & Gessler, 1994): annual temperature, annual minimum temperature, annual maximum temperature, maximum temperature – warmest period, minimum temperature – coldest period, temperature annual range, annual precipitation, precipitation – wettest period, precipitation – driest period and length of growing season. Data for a final variable, average potential evapotranspiration (PET), were also obtained from the CFS using the simplified Penman–Monteith formulation by Hogg (1994).

### Structure of genetic diversity

For the three subsets we wanted to understand the contributions of specific loci to genetic differentiation among sites through redundancy analysis (RDA). We transformed the allele frequencies per site using the Hellinger transformation (Legendre & Gallagher, 2001) using the ‘decode’ function in the vegan package in R (Oksanen *et al.*, 2013), and used the transformed data in RDA using the ‘rda’ function (also part of the vegan package). We looked for outlier loci within the top 10% of loci loading on the first and second principal component axes.

The correlation between genetic diversity and the environment can be the result of a causative process, or the result of shared spatial processes (Borcard *et al.*, 1992). Therefore, we need to be able to separate the contributions of the environment and spatial heterogeneity from their shared contribution to explaining the variation in genetic diversity. To do this, we performed variance partitioning (Borcard *et al.*, 1992; ter Braak & Verdonschot, 1995) using multiple functions in the vegan package. With this approach we used allele frequencies for each locus at our sampling sites to represent genetic diversity, and performed the partitioning using the ‘varpart’ function. This approach calculates the variance in the allele frequencies that is explained by the environment, spatial heterogeneity and their shared contribution and provides adjusted *R*^2^ values for each component. Following that we performed RDA for the models followed by ANOVA to test for significance using permutation. For each data set we reduced the environmental variables to include only those that were not correlated above 0.80. We performed variance partitioning for all three data sets.

### Outlier detection

Four complimentary methods were used to detect outliers. First, we used the approach of Excoffier *et al*. (2009) implemented in arlequin which identifies outliers by comparing the levels of genetic diversity and differentiation between populations. This method is based on the calculation of Beaumont & Nichols (1996). Refinements in arlequin include more flexibility for the mutation model, calculation of heterozygosity to account for different mutation rates, and an option to estimate outliers with either an infinite island or hierarchical demographic model. We estimated the number of putative outliers for all three subsets using both demographic models. For the hierarchical model, we split the pure species data into lodgepole and jack pine groups, and the lodgepole pine data were split into north and south and jack pine into east and west based on population structure analysis (Table 1; Cullingham *et al.*, 2013). Loci were considered to be significant outliers if *P* ≤ 0.01.

Secondly, we used the Bayesian method of Foll & Gaggiotti (2008) implemented in bayescan. Outliers were identified by the direct estimation of a posterior probability for each locus using a reversible-jump Monte Carlo Markov chain (MCMC). For each data set we calculated outliers three times to ensure robustness, each time using a burn-in of 10 000 iterations, a thinning interval of 50, and a sample size of 10 000. We ran different values of the prior odds of the neutral model to quantify how this affected our results (Lotterhos & Whitlock, 2014). We set this to four different values: 10, as suggested in the manual; the number of loci in the data set; 1000; and 10 000. We completed three replicates for each parameter set, and included only those loci that were identified across all three runs for each parameter set. Significance of loci was tested using a maximum false discovery rate of 0.05.

Thirdly, we considered the effects of the environment on locus-specific differentiation directly using an individual-based analysis that estimates spatial coincidence (Joost *et al.*, 2007) implemented in matsam. This method was originally designed for amplified fragment length polymorphism (AFLP) data, but can be used for SNP data (Pariset *et al.*, 2009). Here, a logistic regression model is implemented where individuals are coded with the presence/absence of an allele, and the association between the allele and the environmental parameters is measured across sites. We considered the model fit to be significant when both the G and Wald tests were significant following Bonferroni correction at a 99% confidence level (Joost *et al.*, 2007).

Fourthly, we used a method to investigate environmental correlations that also considers population structure (Coop *et al.*, 2010). To do this, we used bayenv to develop a correlation matrix of neutral loci using SNPs that were unlinked and had not been identified by any of the outlier methods. We calculated the correlation matrix multiple times and compared the outputs in R to ensure it was well estimated. We then ran three iterations for each SNP against the 11 environmental variables and three geographical variables (longitude, latitude and elevation) with 50 000 MCMC steps using a batch file to estimate the BayesFactor. We used Jeffreys' scale of evidence (Jeffreys, 1961) to interpret the BayesFactor; therefore, we assigned levels of support for locus × environment associations as follows: BayesFactor 3–10, substantial support; 10–30, strong support; 30–100, very strong support; and > 100, decisive support. Our final list was comprised of those locus × environment associations with supported BayesFactors across iterations. We used binomial *t*-tests to assess whether there were differences in the number of significant SNPs found for the environmental variables between lodgepole and jack pine.

### SNP annotation

We compared the contig sequences of the candidate outlier SNPs to the most recent release of the loblolly pine genome (*Pinus taeda* assembly v1.01; http://pinegenome.org/pinerefseq/) using gmap (Wu & Watanabe, 2005; Wu & Nacu, 2010). The loblolly pine is the closest relative to our species with a full draft genome. The program gmap identifies intron/exon boundaries and indicates the amino acid sequence based on the open reading frames. Using this information we were able to identify whether the SNPs were intragenic and if they resulted in amino acid changes. We used these data to further refine our outlier candidate list, where synonymous substitutions may not represent true positives.

### Genomic clines

Finally, we estimated the genomic clines of the SNP loci using introgress (Gompert & Buerkle, 2010). This R package implements a method that estimates the genomic cline of each locus. First, parental allele frequencies are calculated; these are then used to estimate the hybrid index for all hybrid populations. Finally, multinomial regression functions are performed to estimate whether genotypes at each locus are within the neutral model expectation based on an individual's hybrid index. Significance testing of this cline indicates whether the locus is behaving based on neutral expectations or if an allele is introgressing more or less frequently than expected. We used the parametric option for estimating the significance for each cline as our loci did not meet the expectation of similar allele frequencies across parental populations (Gompert & Buerkle, 2009). We used an adjusted *P*-value through a false discovery rate of 0.05, implemented in the qvalue R package.

## Results

Observed heterozygosity ranged from 0.00 to 0.90 for both lodgepole and jack pine. This resulted in fixation indices ranging from −0.80 to 0.61 for lodgepole pine and −0.81 to 0.38 for jack pine. Those loci with extreme heterozygosity and fixation indices were not common (10 loci in lodgepole, and 10 loci in jack pine) and exhibited extreme values for one of the two species, but not both (Supporting Information Table S1). There was no evidence of null alleles for these loci based on the proportion of missing data. We identified 24 linkage groups in lodgepole pine and 20 groups in jack pine; nine of these were shared between the species. The number of loci in the linkage groups ranged from two to four (Table S1). Thirteen of the linkage groups were locus pairs that occurred on the same contig. Interestingly, there were an additional 11 SNP locus pairs that were not linked but were characterized from the same contig. While the inclusion of linked loci may create a small bias for some of the outlier detection methods we were using, we decided to include all of them in the outlier analysis. Linkage indicates correlated allele frequencies regardless of physical linkage; therefore the linked loci may be linked statistically but not physically if they are under the same evolutionary pressures (Howe *et al.*, 2003; Tsumura *et al.*, 2012). Given that physical linkage in conifers declines within the length of an average-sized gene (Neale & Savolainen, 2004), many of our linkage groups probably reflect statistical linkage. Also, for physically linked loci, there was no means for us to identify which locus is under selection. Therefore, by identifying both loci, we could characterize them further and increase the likelihood of identifying relevant adaptive variation.

We successfully annotated most contigs through sequence similarity with informatively annotated sequences from other species. Fifty contigs showed sequence similarity to either Arabidopsis or white spruce sequence annotated as having unknown function, and six of the contigs showed insufficient similarity to sequences in the databases that were queried (Table S1). Annotation for the remaining contigs included a wide range of proteins involved in an assortment of molecular processes with > 90 contigs falling under cellular processes, plastid proteins, biosynthetic pathways, membrane function and response to stress categories.

### Structure of genetic diversity

The amount of genetic variation that partitioned the sites estimated using RDA varied among the three data sets. The first two axes of the RDA for lodgepole pine explained 40.17% of the variation among the sites (Table 2). Of the top 10% of loci loadings on the first axis, three outliers were found (Lodgec118p350, Jp_c13093p749 and Jp_c13875p1478). An additional six loci exhibited loadings falling within the top 10% for both axes (Lodgec1087p211, Lodgec2304p514, Jp_c23409p668, Jp_c44933p494, JpLpc44782p470 and Jp_c43213). For jack pine, the first two axes of the RDA explained 79.88% of the variation; three of the four outliers loaded on the first axis (Lodgec6568p554, Jackc1504p209 and Jp_c28904p670). For the combined data set, axes 1 and 2 explained 93.25% of the variation. Five outliers had high loadings on the first axis (JpLpc66545p1207, JpLpc33893p239, JpLpc41319p340, Lp_c00150p459 and JpLpc47089p1831), one locus had high loadings on both axes (JpLpc63491p915), and one locus had high loadings on the second axis (Lodgec118p350).

**Table 2:** Adjusted *R*^2^ and associated *P*-values for variance partitioning of allele frequency data for lodgepole pine (*Pinus contorta* var. *latifolia*), jack pine(*Pinus banksiana*) and the combined subset

	Lodgepole pine		Jack pine		Combined data	
Adj. *R*^2^	*P*-value	Adj. *R*^2^	*P*-value	Adj. *R*^2^	*P*-value
ENV + shared	0.2026	0.010	0.3637	0.044	0.5500	0.005
GEO + shared	0.2307	0.005	0.3192	0.220	0.7455	0.005
ENV	0.0422	0.330	na		0.1093	0.022
GEO	0.0703	0.350	na		0.3047	0.005
Shared	0.1604		na		0.4407	
Unexplained	0.7271		0.6363		0.1453	

The effects of environmental variables (ENV) and spatial coordinates (GEO) on genetic diversity among sites were estimated seperately, as well as the shared effects on the data. Individual components for jack pine could not be estimated (na) as a result of correlation between ENV and GEO.

The shared and separate contributions of environment and spatial heterogeneity in explaining the variation in genetic diversity for each data set were examined (Table 2). For lodgepole pine we retained the following environmental variables following correlation analysis: elevation, maximum temperature – warmest period, temperature annual range, precipitation – wettest period, precipitation – driest period, annual minimum temperature and annual maximum temperature. Neither the environmental variables nor the geographical variables explained a significant proportion of the variation on their own. However, there was shared variation between the variables that explained a significant amount of variation in the allele frequency data (adjusted *R*^2^ = 0.1604; *P* < 0.01). For jack pine, the environmental variables were highly correlated with each other, and with geography. Only elevation was retained in the final model, and the variation in genetic data explained by this was almost entirely shared with the variation explained by geography (adjusted *R*^2^ = 0.3637). For the combined subset, environmental variables were reduced to: elevation, average PET, minimum temperature – coldest period, precipitation – wettest period, precipitation – driest period, and annual temperature range. We found that both the environment and spatial heterogeneity independently contributed significantly to explaining allele count variation among sites (adjusted *R*^2^ = 0.1093 and 0.3047, respectively), and a large proportion of the variation in genetic diversity was explained by both processes (adjusted *R*^2^ = 0.4407).

### Outlier detection

**Figure 1:**
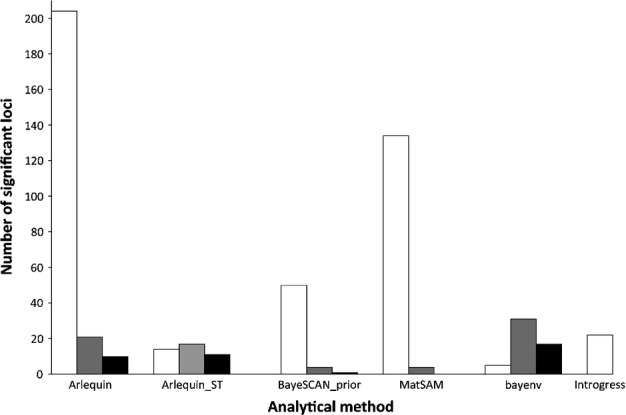
Comparison of the number of outlier loci identified using seven different approaches on three data sets: species data (white bars), pure lodgepole pine (*Pinus contorta* var. *latifolia*; grey bars), and pure jack pine (*Pinus banksiana*; black bars). The methods used were the island and hierarchic demographic models in arelquin (Arelquin and Arelquin_ST, respectively), bayescan with and without prior odds (only the BaySCAN_prior is shown here), matsam, bayenv and introgress.

We identified 21 and 17 outliers in lodgepole pine using the island and hierarchical demographic models in arlequin, respectively (Fig. 1). Fourteen loci were identified in both models; of these, all but two loci exhibited signals of positive selection. We identified 10 and 11 outliers in jack pine using the island and hierarchical models, respectively. Only three of these loci were shared between the models at the 0.01 significance level, while an additional four loci were shared at the 0.05 level. The three commonly identified loci had signals of positive selection. For the combined subset, we identified 204 and 14 loci using the island and hierarchical models, respectively. All 14 loci identified with the hierarchical model were also identified with the island model. Six of the 14 loci had signals of negative selection. One locus was identified in both jack pine and the combined subset, but no loci were identified across all subsets.

**Figure 2:**
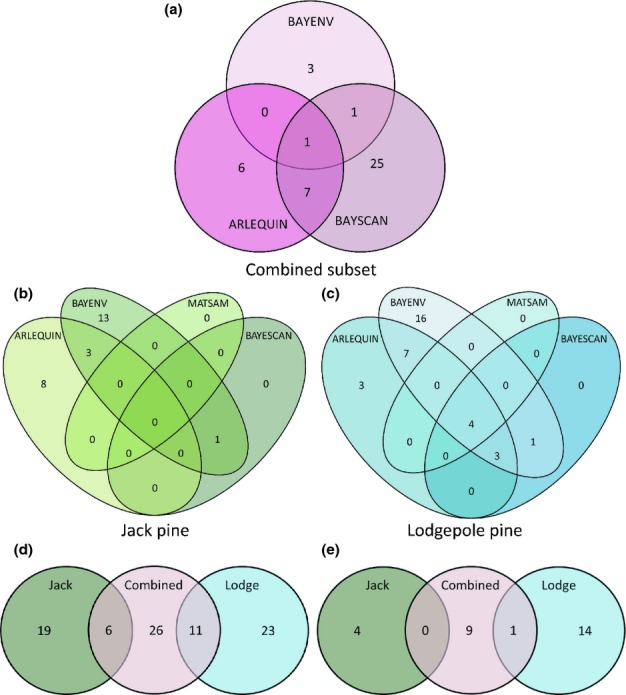
Venn diagrams illustrating the overlap in outlier detection across the three data sets for the different outlier detection methods, and among the data sets overall: (a) combined subset, (b) pure lodgepole pine (*Pinus contorta* var. *latifolia*), (c) pure jack pine (*Pinus banksiana*), and (d) across the data sets before filtering based on consensus (e) consensus set of outliers. We used the results for the hierarchical model for arelquin and the prior odds for the bayscan results.

Using bayescan we consistently identified nine loci within lodgepole pine across all iterations. We identified four of these nine loci with the prior odds set to the total number of loci detected, and three of these loci for 1000 and 10 000 prior odds (Fig. 1). All of these exhibited a signature of positive selection. We chose to use the set of nine loci because all of these belonged to the set of common SNPs also detected using the other outlier detection methods (Fig. 2). We identified only one locus showing positive selection in jack pine using both the default parameters and the adjusted prior odds, and no loci using 1000 or 10 000 prior odds. For the combined subset we detected 116 loci using the default parameters, 50 loci using the number of loci prior odds, 34 loci at a prior odd of 1000, and only six loci with a prior odd of 10 000. The loci identified for each of these sets were also identified using the default parameters. We chose to use the 34 loci identified using the prior of 1000 as there was greater overlap for this set with the other outlier detection methods, as described below. Of these 34 loci, 11 had signatures of negative selection. Only one locus was identified in common between the lodgepole pine and the combined subsets.

We identified four loci in lodgepole pine that were significantly correlated to environmental variables following Bonferroni correction for both the G and Wald tests using matsam. We did not identify any environmental correlations in the jack pine subset. For the combined subset we identified 134 loci with significant environmental correlations. Two of the loci identified in lodgepole pine were also identified in the combined subset.

We identified 31 loci in lodgepole pine with environmental correlations using bayenv. For the jack pine subset we identified 17 loci, and only five loci in the combined subset. Three loci were found in common between the lodgepole pine and combined subsets. Most of the loci identified across all subsets were associated with more than one environmental variable (Table 3). We found that the proportion of associations with the environmental variables was statistically different between lodgepole and jack pine based on two-sample binomial *t*-tests for minimum temperature – coldest period (lodgepole > jack pine; *t* = 4.472; *P* < 0.001), precipitation – driest period (lodgepole > jack pine; *t* = 6.263; *P* < 0.001), longitude (jack > lodgepole pine; *t* = 3.216; *P* = 0.001), maximum temperature – warmest period (jack > lodgepole pine; *t* = 7.343; *P* < 0.001), and average PET (jack > lodgepole pine; *t* = 3.578; *P* = 0.001) following Bonferroni correction (α = 0.004).

**Table 3:** Single nucleotide polymorphism (SNP) loci identified as adaptive candidates by correlation to environmental variables using two different data sets: pure lodgepole pine (*Pinus contorta* var. *latifolia*), and pure jack pine (*Pinus banksiana*); Jeffreys' scale of evidence is indicated for the SNP × environmental variable combination from the BayesFactors calculated in bayenv (substantial (Subst.), 3–10; strong, 10–20; very strong, 30–100; decisive, > 100)

Locus	Data	Longitude**	Latitude	Elevation*	Temp AnnRang*	Ann Temp	Ann Min Temp	Ann Max Temp	MaxTemp WarmPer**	MinTemp ColdPer**	Length GrowSeas	Average PET**	Ann Precip*	Precip DryPer**	Precip WetPer
Jackc1410p116	Lodgepole	Strong			Strong					V. strong			Subst.		
Jackc406p342	Lodgepole	Subst.			Subst.					Subst.					
Jp_c13093p749	Lodgepole		Strong	Decisive	V. strong					V. strong				Subst.	
Jp_c13875p1478	Lodgepole			Subst.	V. strong					V. strong			Strong	Subst.	
Jp_c20590p588	Lodgepole	Subst.			Subst.				Subst.				Subst.		
Jp_c23409p668	Lodgepole		Subst.	V. strong	V. strong					Decisive			Subst.	Subst.	
Jp_c24821p611	Lodgepole		Strong	Decisive	V. strong					V. strong			Subst.	Strong	
Jp_c26537p1091	Lodgepole		Subst.	Strong											
Jp_c28163p1406	Lodgepole			V. strong	V. strong					V. strong			Subst.	Strong	
Jp_c28993p897	Lodgepole	Subst.		Strong	V. strong				Subst.	V. strong			Subst.	Subst.	
Jp_c43213p458	Lodgepole	Subst.		Subst.	Strong					Strong					
Jp_c44637p536	Lodgepole	Subst.	Subst.	V. strong	Decisive	Subst.				Decisive			Subst.	Subst.	
Jp_c44933p494	Lodgepole	Subst.		V. strong	V. strong					V. strong				Subst.	Strong
JpLpc15608p1838	Lodgepole				Subst.					Subst.			Subst.	Subst.	
JpLpc21364p981	Lodgepole		Decisive	Decisive	Strong					V. strong	Subst.		Subst.	V. strong	
JpLpc31990p707	Lodgepole			Subst.	Strong					Strong				Subst.	
JpLpc31990p90	Lodgepole			Subst.	Subst.									Subst.	
JpLpc39993p867	Lodgepole	Subst.													
JpLpc41319p340	Lodgepole				Subst.					Subst.					
JpLpc44494p452	Lodgepole	Subst.			Subst.										
JpLpc44782p470	Lodgepole	Subst.		Strong	V. strong	Subst.		Subst.		V. strong					
JpLpc45225p571	Lodgepole			Subst.	Strong					V. strong					
JpLpc63491p915	Lodgepole			Strong											
JpLpc72792p1173	Lodgepole			Subst.	Subst.					Subst.					
JpLpc79147p1879	Lodgepole					Strong		Strong							
Lodgc1087p211	Lodgepole		V. strong	Subst.				Subst.				Subst.			
Lodgc118p350	Lodgepole		V. strong	Strong										Subst.	
Lodgc2304p514	Lodgepole		V. strong	Strong											
Lodgc2413p445	Lodgepole		Subst.	Strong											
Lp_c04318p2154	Lodgepole		V. strong	Strong								Subst.			
Jackc1504p209	Jack		Strong			Subst.	Subst.	Subst.				Subst.	Subst.		
Jp_c28904p670	Jack	Subst.	Strong			Subst.		Subst.	Subst.			Subst.	Subst.		
Jp_c34152p781	Jack	Subst.	Subst.					Subst.							
JpLpc00337p904	Jack	Subst.		Subst.	Subst.				Subst.		Subst.				
JpLpc32323p740	Jack	Strong	Strong					Subst.	Strong			Subst.			
JpLpc47089p1831	Jack	Subst.	Subst.						Subst.						
JpLpc54814p513	Jack			Subst.	Subst.				Subst.	Subst.					
JpLpc66545p1207	Jack	Subst.		Subst.					Strong			Subst.			
JpLpc79197p531	Jack	Subst.							Subst.			Subst.			
JpLpc86157p398	Jack	Strong		Subst.	Subst.				V. strong		Subst.	Subst.			
JpLpc86157p76	Jack	Subst.		Subst.	Subst.				Strong		Subst.	Subst.			
Lodgc4407p669	Jack			Subst.	Subst.				Subst.						
Lodgc6568p554	Jack	Subst.		Subst.	Subst.				Strong		Subst.	Strong			
Lp_c01813p1225	Jack						Subst.							Subst.	
Lp_c08410p197	Jack	Subst.							Subst.						
Lp_c14699p706	Jack	Subst.							Subst.			Subst.			
Lp_c31229p1539	Jack	Subst.	Subst.						Subst.						

** , Significantly greater number of correlations for jack pine to that environmental variable than for lodgepole pine;

* , significantly greater number of correlations for lodgepole pine to that environmental variable than for jack pine.

Temp AnnRang, temperature annual range; Ann Temp, annual temperature; Ann Min Temp, annual minimum temperature; Ann Max Temp, annual maximum temperature; MaxTemp WarmPer, maximum temperature during the warmest period; MinTemp ColdPer, minimum temperature during the coldest period; Length GrowSeas, length of the growing season; Average PET, average potential evapotranspiration; Precip DryPer, precipitation during the driest period; Precip WetPer, precipitation during the wettest period.

### Summary of the outlier detection

Within each data subset, we compared the results obtained using the four different detection methods using Venn diagrams to determine the degree of overlap (Fig. 2). In lodgepole pine, seven loci were identified across three methods, seven loci were identified across two methods, and 19 were identified by only one of the methods (Fig. 2c). In jack pine, four loci were identified using two methods, and 21 loci were identified with one method (Fig. 2b). Across the combined subset we did not consider the results from matsam; in this case, one locus was detected across the three other methods, eight loci were detected across two methods, and 34 loci were detected using a single method (Fig. 2a).

We also compared the overlap of detected loci across the three subsets of data, again using a Venn diagram (Fig. 2d). We used the results from the hierarchical demographic model from arlequin and discarded the results from matsam for the combined subset. There were no loci that were shared between lodgepole and jack pine, but 24% of the loci in jack pine and 32% of the lodgepole loci were shared with the species data (Fig. 2), resulting in the identification of 85 putative candidate loci. If we consider the consensus for each subset of data and only identify loci with at least two significant tests, we reduce our candidate list to 28 loci (Fig. 2e), 15 exclusive to lodgepole pine, four exclusive to jack pine, eight exclusive to the combined subset, and only one shared between lodgepole pine and the combined subset (Table 4).

**Table 4:** Summary of putative adaptive candidates detected across multiple methods in at least one of three subsets of data: lodgepole pine (*Pinus contorta* var. *latifolia*), jack pine (*Pinus banksiana*), and combined, including diversity measures (observed heterozygosity (H_O_), expected heterozygosity (UH_E_), and fixation index (F)), their estimated linkage group, the number of positive outlier tests for each data set, the direction of selection, the putative amino acid (AA) change (I, intragenic; NC, noncoding; NS, nonsynonymous; S, synonymous), and their putative annotation

Locus	Lodgepole pine				Jack pine				Linkage group	Outlier detection			Introgress *P*-value	Direction	AA change	Annotation
	*N*	Ho	UHe	F	*N*	Ho	UHe	F		Combined	Lodgepole	Jack				
JpLpc45225p571	368	0.043	0.043	−0.022	99	0.202	0.183	−0.112	LP07	1	3		0.002	Positive	NS	B-cell receptor-associated protein 31-like
Lodgc1087p211	368	0.239	0.270	0.112	100	0.000	0.000		LP13	1	3		0.005	Positive	NS	Proteasome alpha type 7
Lodgc2304p514	367	0.240	0.274	0.123	100	0.010	0.010	−0.005	LP14	1	3		0.240	Positive	NS	Triosephosphate isomerase
Jp_c01249p362	349	0.610	0.425	−0.439	93	0.516	0.466	−0.113	JP03	2			**0.000**	Negative	NC	Short-chain dehydrogenase-reductase B/KR domain
Jp_c21224p439	368	0.402	0.381	−0.057	100	0.350	0.351	−0.004		2			0.547	Negative	S	Transketolase/dehydrogenase-5-phosphate synthase
Jp_c25075p377	368	0.416	0.419	0.005	100	0.560	0.451	−0.248		2			**0.000**	Negative	S	Phenylcoumaran benzylic ether reductase/ NmrA-negative transcriptional regulator
JpLpc33893p239	368	0.016	0.016	−0.008	100	0.070	0.104	0.327		3			0.585	Positive	NC	Auxin-F-box protein 5
JpLpc41319p340	368	0.027	0.032	0.153	100	0.030	0.049	0.385		2	1		0.992	Positive	NC	Uncharacterized BCR, YbaB family COG0718
JpLpc47089p1831	366	0.016	0.016	−0.008	100	0.190	0.173	−0.105		2		1	0.249	Positive	NC	Dof-type zinc finger DNA-binding family protein
JpLpc63491p915	368	0.019	0.019	−0.010	100	0.240	0.256	0.059		2	1		0.002	Positive	NC	Cytokinin response factor/AP2 domain
JpLpc66545p1207	368	0.019	0.019	−0.010	99	0.081	0.078	−0.042		2		1	0.020	Positive	S	Transcribed locus
Lp_c00150p459	367	0.014	0.014	−0.007	100	0.130	0.173	0.244		2			0.353	Positive	I	Circadian clock associated 1
Lodgc118p350	365	0.427	0.467	0.083	100	0.010	0.010	−0.005	LP13	2	3		**0.000**	Positive	S	Sedoheptulose-bisphosphate/fructose-1-6-bisphosphate – chloroplast like
Jp_c13093p749	364	0.025	0.024	−0.013	100	0.550	0.496	−0.113	LP04/JP11		2		0.001	Positive	NC	HSP20-like chaperones superfamily protein
Jp_c13875p1478	367	0.071	0.068	−0.037					LP05		2		na	Positive	S	Pectin methylesterase
Jp_c23409p668	368	0.024	0.024	−0.012	100	0.560	0.499	−0.127			2		0.205	Positive	S	Glyoxalase/bleomycin resistance protein/dioxygenase superfamily
Jp_c24821p611	368	0.027	0.027	−0.014	100	0.320	0.284	−0.134		1	2		0.494	Positive	S	Nodulin MtN3 family protein
Jp_c43213p458	368	0.019	0.019	−0.010	100	0.900	0.499	−0.812	LP07		2		**0.000**	Positive	S	Lipid transfer protein/seed storage 25 albumin superfamily protein
Jp_c44637p536	368	0.022	0.022	−0.011	100	0.470	0.502	0.059	LP07		2		0.579	Positive	NC	Coatomer alpha subunit/glycine-rich protein
Jp_c44933p494	368	0.024	0.024	−0.012	100	0.390	0.410	0.043		1	2		0.139	Positive	S	Photosystem II reaction center protein
JpLpc15608p1838	337	0.151	0.140	−0.082							3		na	Positive	NC	Transducin family protein/WD-40 repeat family protein
JpLpc21364p981	368	0.052	0.056	0.069	100	0.140	0.131	−0.075		1	3		**0.000**	Positive	S	myb-like DNA binding domain
JpLpc44782p470	368	0.022	0.022	−0.011	100	0.320	0.297	−0.084		1	2		0.335	Positive	S	KNOX1/2 domain/KNOTTED-like
Lp_c04318p2154	368	0.207	0.215	0.038	100	0.000	0.000		LP14		3		0.267	Positive	NC	Carbohydrate binding molecule/starch branching enzyme 2.2
Jackc1504p209	368	0.003	0.003	−0.001	100	0.300	0.309	0.025				2	0.002	Positive	S	Transcribed locus
Jp_c28904p670	368	0.011	0.011	−0.005	100	0.350	0.488	0.279				2	**0.000**	Positive	**NC**	Ubiquitin-conjugating enzyme 28//E3 interaction residues
Jp_c34152p781	347	0.127	0.124	−0.024	99	0.515	0.502	−0.031				2	0.018	Positive	S	Ribosomal protein L27
Lodgc6568p554	368	0.329	0.366	0.100	100	0.100	0.131	0.232	JP09			2	0.010	Positive	S	Flavanone 3-hydroxylase

Bold values indicate *P*-values that are significant following correction for FDR. na, not available.

### SNP annotation

Among 28 candidate loci, all aligned with high sequence similarity to the loblolly pine draft genome. We were able to identify open reading frames and, from this, identify whether the SNPs resulted in amino acid changes. We found three of our SNPs to represent nonsynonymous mutations, 14 represented synonymous mutations, 10 represented variation in the 5′ or 3′ untranslated region, and one represented an intragenic variant (Table 4). While the majority of SNPs were developed from a transcription assembly, the intragenic SNP among our candidates was identified through resequencing as part of our SNP validation for the bead-chip development (Cullingham *et al.*, 2013).

### Genomic clines

Based on the genomic clines estimated in introgress, we identified 38 loci that exhibited inheritance patterns that deviated significantly from neutral expectations after estimating the false discovery rate (Table S1). The genomic cline patterns were complex and diverse, but included evidence of lodgepole introgression into jack pine (LL+, JJ−) and vice versa, as well as evidence of heterozygote advantage (LL−, LJ+, JJ−), positive selection (LL+, LJ−, JJ+; LL, LJ−, JJ), and negative selection (LL−, LJ+, JJ−; LL, LJ+, JJ). Of the significant loci, six were consensus outliers: two were identified in the lodgepole pine subset and of these one was identified in the combined subset. An additional locus was identified in just the combined subset, and one in jack pine (Table 4).

## Discussion

Through the application of multiple contrasting methods, we have identified candidate genes representing putative adaptive variation both within and between lodgepole and jack pine. As expected, loci for which we detected signatures of selection within the species subsets were not shared, potentially as a result of contrasting environmental adaptations. A high number of synonymous substitutions suggest that, despite a stringent approach to identifying candidates, a proportion may still represent false positives. The roles of regulatory, noncoding regions as a source of adaptive variation are implicated given that a large number of our candidate loci were found in the 3′ and 5′ untranslated regions.

The performance of outlier detection methods has been reviewed using simulated data (Excoffier *et al.*, 2009; Pérez-Figueroa *et al.*, 2010; Narum & Hess, 2011; De Mita *et al.*, 2013); however, those studies varied in the methods they assessed in comparison to our approach. Therefore, we provide a brief commentary on performance using empirical data. Our reasoning for using multiple methods is twofold. First, we expected the two classes of method to identify different sets of outliers (Vasemägi & Primmer, 2005; Eckert *et al.*, 2010a; Nunes *et al.*, 2011) and, secondly, the consensus among multiple methods has been suggested as a more robust means to reduce false positives (Narum & Hess, 2011; Nunes *et al.*, 2011; Gosset & Bierne, 2012; De Mita *et al.*, 2013). Nevertheless, we recognize that it is inherently difficult to identify which outlier loci are false positives and future work connecting these to phenotype will be required.

Our analyses demonstrate the impact that population demography can have on the detection of outliers. Using arelquin and matsam, for the combined subset, resulted in over-estimation of outliers (Fig. 1). Both Nei & Maruyama (1975) and Robertson (1975) recognized that population structure can impact the variance distribution of loci, making the identification of true outliers difficult. We accounted for population structure by using the hierarchical model in arlequin, resulting in a more conservative estimate of putative outliers, which is likely to reflect a more reasonable approximation (Thornton & Jensen, 2007; Bierne *et al.*, 2013). With matsam, there is no means to account for population demographics, and the processes of drift and gene flow can result in genetic clines resulting in a high proportion of false positives (Kimura & Maruyama, 1971; Vasemägi, 2006; Eckert *et al.*, 2010b). Indeed, the difference in the number of detected loci between bayenv and matsam was quite large, prompting us to discard the results from the combined subset analysis using matsam.

The prior odds for the neutral model in bayescan represents how much more likely this model is over the selection model; for example, a prior odds of 1 assumes that they are equally likely. The manual suggests setting the prior odds to 10 for data with a few hundred loci, and therefore we ran the model with the suggested value of 10 then, following Lotterhos & Whitlock (2014), we set the prior equal to the number of loci being tested, and also to 1000 and 10 000. The rationale of a larger prior is to reduce the number of false positives. Increasing this parameter was effective in producing a reasonable list for the combined subset. The recommended value of 10 resulted in 30% of loci being identified as outliers; an estimate well outside the range of other studies (5–10%; Stinchcombe & Hoekstra, 2007), suggestive of a high proportion of false positives. However, at a prior of 10 000, only six loci were detected, which may be too conservative an estimate. Because we are developing a candidate list, we choose a balance that limits Type I and II error by considering the results using a prior of 1000; this approach identified 34 candidate loci, nine of which were identified by other methods (Fig. 2). For lodgepole and jack pine, the default parameter resulted in a reasonable number of outliers; nine and one, respectively. Those nine outlier loci identified in lodgepole pine were also identified as outliers across multiple detection methods (Fig. 2), suggesting that these are good candidates.

The contrasting results between the combined subset and the pure species analyses relates to their underlying population structure. The combined subset has a very strong hierarchy while there is limited population structure within lodgepole and jack pine (Cullingham *et al.*, 2013). Greater population structure will lead to a higher variance (Nei & Maruyama, 1975; Robertson, 1975), which will result in identifying a greater proportion of false positives (Excoffier *et al.*, 2009; Siol *et al.*, 2010; Bierne *et al.*, 2013). The value for the prior odds parameter in bayescan is rarely reported in the literature (e.g. Nielsen *et al.*, 2009; Bradbury *et al.*, 2010; Freedman *et al.*, 2010) but is clearly an important consideration dependent on genetic structure. Future analyses should report the parameter value used, and adjust this parameter accordingly.

Among our candidate outliers, five of them contributed to the variation explained by the first axis for the combined subset of our RDA analysis. Bierne *et al*. (2003) demonstrated that the first axis represents the gradient of introgression, which is correlated with environmental variation. Because of this, environmentally associated outliers that load on this first axis probably represent false positives. None of our candidate loci found for the combined subset were environmentally associated, and therefore the outliers that load on the first axis may represent important adaptations within lodgepole and/or jack pine. Within each of the species we did not find the same significant relationship with the environment, indicating that population structure at the species level is not driven by environmental differences. This suggests two things. First, environmentally associated outliers identified may represent adaptive variation and not false positives. Secondly, these species have been subject to different evolutionary pressures and therefore environment plays an important role in maintaining species differences. Indeed, distributional modelling demonstrates that genetic ancestry of individuals is well predicted by environmental variables (Cullingham *et al.*, 2012). Taken together, the above suggests that there is evidence of environmental adaptation for each of these species. Differences in moisture and temperature tolerances may be the main drivers of adaptive differences. This can have important consequences for mountain pine beetle spread risk. Under environmental stress, for example, drought, lodgepole pine has reduced capacity to defend against attack (Lusebrink *et al.*, 2011; Arango-Velez *et al.*, 2014). If jack pine has a higher tolerance to water limitation, it may be better able to defend under water stress conditions. However, jack pine may have lower tolerance to increased temperature, and therefore jack pine may be more susceptible in future climates based on current warming predictions (Mbogga *et al.*, 2009).

Among our three data sets, the proportion of outlier loci detected ranges from 1% to 4%; if one considers the large size of the *Pinus* genome (Wakamiya *et al.*, 1993), this would translate into thousands of adaptive sites. This observation is counter to the theory of neutral molecular evolution (Kimura, 1968, 1983), and, while there is mounting evidence demonstrating significant levels of Darwinian natural selection (Ellegren, 2008; Hahn, 2008; Frankham, 2012), evidence of adaptive evolution for the majority of plant species (including some conifers) are not significantly different from zero (Grossmann *et al.*, 2010; Eckert *et al.*, 2013). Positive *F*_ST_ outliers can result from other processes aside from local adaptation, including stochastic effects, background selection, and pre- and post-zygotic isolation (see Bierne *et al.*, 2011, 2013). This suggests that some of our outlier loci may not represent true positives, despite our stringent criteria. Based on the mutations of the outlier SNPs estimated from open reading frames, we can refine our candidate list to include what we consider to be true statistical positives. Fourteen of our 28 outliers show synonymous mutations, suggesting that these are neutral. While there is evidence to support the process of natural selection acting on neutral variation through mechanisms such as codon usage bias (Chamary & Hurst, 2004; Carmel *et al.*, 2007), the likelihood that this would create a strong selective signature is small (Akashi, 1999; Yang & Nielsen, 2008). Among our outliers we also observed 11 SNPs that were located within noncoding regions, 10 of which were contained in the 3′ and 5′ untranslated regions. Analyses of plant genomes show limited functional noncoding sequence, indicating little selective pressure on these regions (Lockton & Gaut, 2005; Haudry *et al.*, 2013). However, these were based on angiosperm species with smaller genomes. Among plants, trees with large, outbreeding populations with limited population structure show the greatest evidence of selection and probably have more complex control elements in the untranslated region (Hough *et al.*, 2013). Therefore, these SNP variants may affect expression through processes such as splicing, and transcriptional control (Andolfatto, 2005; Drake *et al.*, 2006). Outlier detection in white spruce also identified a large proportion of putative outlier candidates in noncoding regions (Namroud *et al.*, 2008). Finally, we identified three candidates with nonsynonymous mutations; two of these are involved in regulation, while one is involved in the glycolytic pathway.

Hybridization between species is considered an important source of novel genetic variation for adaptation to act on (Stebbins, 1959; Knobloch, 1972). For example, Lodgc118p350 shows a pattern of jack pine introgression and is also an outlier in the combined subset and lodgepole pine, and Jp_c28904p670 shows a pattern of lodgepole introgression and is a significant outlier in jack pine. While we were expecting to find a greater portion of overlap between significant outliers and genomic clines, we found limited overlap. Hamilton *et al*. (2013) and Luttikhuizen *et al*. (2012) took a similar approach across a hybrid zone and they found little to no overlap between outliers and introgressed loci. Those loci not identified as outliers could still represent important variation that adaptation may act on, especially given changing environments (Campbell *et al.*, 2009).

### Conclusions

The number of consensus outliers that we detected for each data set is below the range found in other studies (5–10%; Stinchcombe & Hoekstra, 2007). The high proportion of outliers in these studies probably represents a high rate of false positives (Nei *et al.*, 2010). False positives are also possible among our candidate loci and could represent stochastic processes or linkage to other candidates, or they may be the result of coupling, where loci under endogenous selection become coupled with exogenous selection which will often coincide with environmental variation (Barton & Hewitt, 1985; Bierne *et al.*, 2011). Given limited genome coverage and a focus on expressed sequences, we have missed adaptive variation in noncoding regulatory regions (Wasserman & Sandelin, 2004). Also, we are missing those loci that have small effects and are not detectable using these methods (Pritchard & Di Rienzo, 2010), although it is expected that adaptation to the environment for these species needs to occur through changes at a small number of loci of large effect given the high level of gene flow, long generation time and large effective population sizes (Howe *et al.*, 2003; Holliday *et al.*, 2010, 2012; Pelgas *et al.*, 2011). Finally, there is the possibility that some of our candidate loci represent false positives. The next steps to identify whether these candidates represent adaptive variation will be, first, to expand our sampling to determine whether we observe the same signal in other populations; secondly, to characterize the function of candidate genes; and, finally, to look at expression profiles of different genotypes, and their response to infection, to identify phenotypic effects, focussing on different environmental conditions.

## Supporting Information

Table S1 Summary of all loci typed in lodgepole pine (Lp) and jack pine (Jp), including diversity measures, estimated linkage group, and outlier summary information
