# Supplementary material for: Cross-species outlier detection reveals different evolutionary pressures between sister species
Source: New Phytol. 2014 Jun 18;204(1):215–29. doi: 10.1111/nph.12896 (PMC4260136; doi:10.1111/nph.12896)
Supplement: Table S1 — Summary of all loci typed in lodgepole pine (Lp) and jack pine (Jp), including diversity measures, estimated linkage group, and outlier summary information [file nph0204-0215-SD1.pdf]

Supporting Information Table S1. Summary of all loci typed in lodgepole (Lp), and jack pine (Jp), including diversity measures (observed heterozygosity (Ho), expected heterozygosity (UHe)), their estimated linkage group, the data subset they were included in ('Data'), the frequency they were detected as an outlier for each subset of data, the direction of selection, and their putative annotation.

Locus	Lodgepole pine				Jack pine				Linkage group	Data	Outlier Detection			Introgress	Direction	Annotation
	N	Ho	UHe	F	N	Ho	UHe	F			Species	Lodge	Jack			
**Jackc1080p157**	368	0.003	0.003	-0.001	100	0.270	0.263	-0.031		species/Jp				0.53		RPM1-interacting protein 4 (RIN4) family protein
**Jackc1237p470**	368	0.082	0.083	0.020	100	0.560	0.495	-0.136		all		1		0.046	Negative	OT U-like cysteine protease
**Jackc1315p459**	368	0.000	0.000		100	0.370	0.339	-0.096		species/Jp				0.523		bZIP transcription factor/ABA-responsive element binding protein
**Jackc1384p110**					100	0.430	0.426	-0.014		Jp						transcribed locus
**Jackc1410p116**	364	0.052	0.051	-0.027	100	0.500	0.495	-0.015		all		1		0.927		postsynaptic protein-related/microtubule-associated protein CRIPT
**Jackc1450p228**	367	0.008	0.008	-0.004	100	0.500	0.422	-0.190		species/Jp				0.08		transcribed locus
**Jackc1483p368**	345	0.006	0.006	-0.003	100	0.410	0.426	0.033		species/Jp				0.044		ubiquitin-specific protease 3
**Jackc149p234**	368	0.003	0.003	-0.001	100	0.270	0.249	-0.089		species/Jp				0.973		4-hydroxy-3-methylbut-2-enyl diphosphate reductase
**Jackc1504p209**	368	0.003	0.003	-0.001	100	0.300	0.309	0.025		species/Jp			2	0.002	Positive	transcribed locus
**Jackc152p517**	368	0.160	0.170	0.056	100	0.390	0.392	-0.001		all				0.788		L-asparaginase/ amidohydrolase
**Jackc1570p273**	367	0.000	0.000		99	0.505	0.496	-0.023		species/Jp				0.325		protein tyrosine kinase superfamily
**Jackc1579p429**					100	0.300	0.322	0.062		Jp						Pleckstrin homology (PH) domain superfamily protein
**Jackc1735p209**	364	0.003	0.003	-0.001	100	0.460	0.444	-0.040		species/Jp				LL+LJ-JJ-		mRNA splicing factor/thioredoxin-like/Dim1 family
**Jackc1859p368**	364	0.088	0.084	-0.046	100	0.480	0.457	-0.055		all				0.788		
**Jackc2307p219**					98	0.224	0.200	-0.126		Jp						DnaJ/Hsp40 cystein-rich domain superfamily protein
**Jackc2433p173**	368	0.003	0.003	-0.001	100	0.480	0.478	-0.009		species/Jp				LL+LJ-; LL-		pentatricopeptide repeat (PPR)/tetratricopeptide repeat (TPR)-like
**Jackc2551p179**					93	0.495	0.493	-0.009	JP01	Jp						WD-40 repeat family protein/beige/BEACH domain-related
**Jackc2623p916**	368	0.008	0.008	-0.004	100	0.560	0.482	-0.167		species/Jp				0.034		transcribed locus
**Jackc2888p563**	368	0.090	0.091	0.010	99	0.374	0.374	-0.003		all				0.604		cytokine-induced anti-apoptosis inhibitor 1, Fe-S biogenesis
**Jackc291p82**					98	0.602	0.487	-0.241		Jp						Heavy metal transport/detoxification superfamily protein
**Jackc3017p105**	368	0.011	0.011	-0.005	100	0.450	0.502	0.100	JP01	species/Jp				0.077		
**Jackc3080p108**	368	0.003	0.003	-0.001	100	0.340	0.309	-0.105		species/Jp			1	0.125	Negative	myb domain protein 3r-4
**Jackc3589p359**					92	0.522	0.488	-0.076		Jp						cytochrome C1 family
**Jackc3789p280**	364	0.003	0.003	-0.001	100	0.240	0.227	-0.061		species/Jp				0.142		transcribed locus
**Jackc391p162**					100	0.290	0.290	-0.004		Jp						photosystem I, PsaA/PsaB protein
**Jackc4003p197**					99	0.414	0.364	-0.144	JP02	Jp						O-acetyltransferase protein/Cas1p-like protein
**Jackc402p99**	368	0.000	0.000		100	0.420	0.457	0.077		species/Jp	1			0	Positive	NADH-ubiquinone oxidoreductase related
**Jackc406p342**	368	0.030	0.035	0.139	100	0.560	0.502	-0.122	JP07	all		1		0.876		heat shock cognate protein 70-1
**Jackc408p413**	365	0.005	0.005	-0.003	100	0.580	0.486	-0.199		species/Jp				0.045		protein of unknown function (DUF3411)
**Jackc452p299**	366	0.003	0.003	-0.001	97	0.361	0.369	0.017		species/Jp				0.531		BEL1-like homeodomain/DNA binding transcription factor
**Jackc473p320**					99	0.485	0.459	-0.061	JP03	Jp						Ribosomal protein L7Ae/L30e/S12e/Gadd45 family protein
**Jackc480p274**	367	0.289	0.274	-0.056	100	0.270	0.263	-0.031		all	1			0.025	Negative	Heavy metal transport/detoxification superfamily protein
**Jackc616p315**					100	0.460	0.457	-0.011	JP04	Jp						septin
**Jackc722p712**	355	0.521	0.494	-0.056	99	0.465	0.491	0.049	LP01/JP05	all				0.037		cinnamoyl-CoA reductase/NAD dependent epimerase/dehydratase
**Jackc816p105**	367	0.011	0.011	-0.005	100	0.330	0.290	-0.143		species/Jp				0.087		
**Jackc833p224**	352	0.548	0.501	-0.097					LP02	Lp						CCCH-type zinc finer family protein
**Jackc915p369**	368	0.329	0.360	0.086	100	0.540	0.490	-0.108	LP03/JP06	all				0.187		ribosomal protein L2 family
**Jackc924p449**					97	0.309	0.317	0.018		Jp						response regulator
**Jackc961p604**	368	0.003	0.003	-0.001	100	0.360	0.414	0.126	JP01	species/Jp				N; LL-,LJ+		protochlorophyllide oxidoreductase A/short chain dehydrogenase
**Jp_c00039p703**	368	0.011	0.011	-0.005	100	0.390	0.382	-0.026	JP02	species/Jp				0.337		adenosine kinase
**Jp_c00242p897**					97	0.629	0.502	-0.260	JP07	Jp						ribosomal protein S5 family protein

Locus	Lodgepole pine				Jack pine				Linkage group	Data	Outlier Detection			Introgress	Direction	Annotation
	N	Ho	UHe	F	N	Ho	UHe	F			Species	Lodge	Jack			
**Jp_c00433p427**	368	0.024	0.024	-0.012	100	0.360	0.422	0.143		all				0.544		GDSL-link lipase/acylhydrolase
**Jp_c00542p351**	368	0.049	0.048	-0.025	100	0.450	0.466	0.029		all				0.172		transcribed locus
**Jp_c00704p802**	367	0.313	0.307	-0.021	100	0.280	0.284	0.008		all				0.004		peroxidase 2
**Jp_c01249p362**	349	0.610	0.425	-0.439	93	0.516	0.466	-0.113	JP03	all	2			0	Negative	short-chain dehydrogenase-reductase B/KR domain
**Jp_c01798p762**					95	0.337	0.309	-0.097		Jp						coatomer gamma subunit putative
**Jp_c01946p291**					100	0.470	0.454	-0.040	JP04	Jp						xyloglucan endo-transflycosylase
**Jp_c01993p623**	364	0.223	0.219	-0.017	99	0.192	0.206	0.065	JPLP01	all				0.628		protein kinase superfamily
**Jp_c01993p67**	367	0.218	0.224	0.024	91	0.099	0.133	0.254	JPLP01	all				0.386		protein kinase superfamily
**Jp_c02124p853**	335	0.069	0.066	-0.036	99	0.202	0.229	0.114		all	1			0.748	Negative	NUP50/nucleoporin
**Jp_c02580p908**	368	0.011	0.011	-0.005	100	0.490	0.496	0.008		species/Jp				0.002		ubiquitin-conjugating enzyme
**Jp_c03167p296**	368	0.024	0.024	-0.012	100	0.290	0.263	-0.107	LP11/JP08	all				0.841		auxin response factor 1/B3 DNA binding domain
**Jp_c03167p428**					99	0.222	0.214	-0.043		Jp						auxin response factor 1/B3 DNA binding domain
**Jp_c03968p478**	368	0.424	0.370	-0.147	100	0.360	0.333	-0.085	LPL1	all				0.044		plasma membrane intrinsic protein 2;8/aquaporin PIP2
**Jp_c03968p72**	368	0.620	0.496	-0.250	100	0.110	0.104	-0.058	LPL1	all				0.02		plasma membrane intrinsic protein 2;8/aquaporin PIP2
**Jp_c04973p670**	343	0.510	0.493	-0.036	100	0.470	0.491	0.038	LP01/JP05	all				0.042		alcohol dehydrogenase/zinc-binding dehydrogenase
**Jp_c05130p655**	368	0.011	0.011	-0.005	100	0.400	0.478	0.159		species/Jp				0.223		voltage dependent anion channel
**Jp_c05171p407**	367	0.104	0.103	-0.005	98	0.643	0.484	-0.336		all				0.063		translocase of outer membrane 22-V
**Jp_c06398p643**	368	0.543	0.501	-0.087	100	0.380	0.367	-0.042	LP02	all				0.003		thylakoid rhondanese-like
**Jp_c06551p422**	368	0.095	0.100	0.052	100	0.540	0.495	-0.096		all				0.92		photosynthetic electron transfer C
**Jp_c07427p1497**	368	0.003	0.003	-0.001	100	0.180	0.197	0.081		species/Jp				0.972		tranlocon outer complex protein/DUF3406
**Jp_c09653p489**	367	0.324	0.358	0.093	100	0.540	0.490	-0.108	LP03/JP06	all				0.071		photosystem II reaction center PSB29 protein/thylakoid formation protein
**Jp_c12031p335**	367	0.387	0.423	0.083	93	0.624	0.454	-0.382		all	1			0	Negative	protein of unknown function (DUF538)
**Jp_c12214p1395**	368	0.071	0.073	0.035	100	0.460	0.482	0.042		all				LL-; LJ-,JJ+		homeobox domain
**Jp_c12920p1329**	366	0.025	0.024	-0.012	100	0.280	0.270	-0.042		all				0.302		inner membrane family protein
**Jp_c13093p749**	364	0.025	0.024	-0.013	100	0.550	0.496	-0.113	LP04/JP11	all		2		0.001	Positive	HSP20-like chaperones superfamily protein
**Jp_c13875p1478**	367	0.071	0.068	-0.037					LP05	Lp		2				pectin methylesterase
**Jp_c15855p785**	368	0.185	0.202	0.086	100	0.310	0.290	-0.074		all	1			0	Negative	A20/AN1-like zinc finger family protein
**Jp_c15992p560**	368	0.014	0.014	-0.007	96	0.219	0.313	0.297		species/Jp				0.061		transducin/WD-40 repeat-like superfamily/homologue of yeast autophagy
**Jp_c17571p991**	368	0.003	0.003	-0.001	100	0.500	0.474	-0.061		species/Jp				0.413		high cyclic electron flow 1/fructose 1-6-bisphosphatase
**Jp_c18278p614**					95	0.368	0.446	0.169		Jp						thioredoxin superfamily protein
**Jp_c18600p510**	353	0.003	0.003	-0.001	99	0.293	0.279	-0.055		species/Jp				0.009		ribosomal protein L32
**Jp_c19293p339**					99	0.404	0.500	0.188		Jp						glutathione s-transerfase/translation elongation factor
**Jp_c20036p1123**	367	0.079	0.076	-0.041	100	0.390	0.361	-0.085		all				0.345		S-formylglutathione hydrolase/esterase
**Jp_c20182p499**	367	0.079	0.076	-0.041	100	0.230	0.263	0.122		all				0.054		transcribed locus
**Jp_c20432p742**	368	0.005	0.005	-0.003	100	0.230	0.235	0.015		species/Jp	1			0.073	Positive	phytoene desaturation 1/4-hydroxyphenylpyruvate/Glyoxalase/Bleomycin
**Jp_c20538p910**	344	0.523	0.481	-0.089	99	0.505	0.502	-0.010		all	1			0	Negative	transcribed locus/glycine rich protein
**Jp_c20590p588**	354	0.045	0.044	-0.023	100	0.480	0.486	0.008		all		1		0.136		RNA -binding protein/RNA recognition motif
**Jp_c20770p419**	368	0.008	0.008	-0.004	100	0.590	0.426	-0.392		species/Jp				0.017		eukaryotic translation initiation factor 2 beta subunit
**Jp_c20964p544**	368	0.076	0.073	-0.040	100	0.350	0.351	-0.004		all				0.793		NAD(P)-binding Rossmann-fold superfamily protein/short chain dehydrogenase
**Jp_c21015p448**	368	0.168	0.154	-0.092	100	0.420	0.414	-0.020		all				0.861		P-loop containing nucleoside triphosphate hydrolase/SRP54-type, GTPase domain
**Jp_c21018p454**	365	0.003	0.003	-0.001	100	0.560	0.502	-0.122		species/Jp				0.201		erythronate-4 phosphate dehydrogenase family protein
**Jp_c21171p1069**	353	0.445	0.485	0.082	99	0.323	0.324	-0.003		all				0.114		S-adenosyl-L-homocysteine hydrolase/NAD binding domain
**Jp_c21224p439**	368	0.402	0.381	-0.057	100	0.350	0.351	-0.004		all	2			0.547	Negative	transketolase/dehydrogenase-5-phosphate synthase

Locus	Lodgepole pine				Jack pine				Linkage group	Data	Outlier Detection			Introgress	Direction	Annotation
	N	Ho	UHe	F	N	Ho	UHe	F			Species	Lodge	Jack			
**Jp_c21306p1347**	367	0.000	0.000		92	0.511	0.459	-0.118		species/Jp				0.816		glyceraldehyde 3-phosphate dehydrogenase of plastid 1
**Jp_c21401p474**	367	0.177	0.171	-0.040	100	0.510	0.494	-0.038		all				0.303		pyridine nucleotide-disulphide oxidoreductase/monodehydroascorbate reductase 1
**Jp_c21871p717**	352	0.020	0.020	-0.010	99	0.414	0.473	0.120		all				0.618		methionine sulfoxide reductase B 2
**Jp_c21979p359**					100	0.320	0.284	-0.134		Jp						ribosomal protein S26e
**Jp_c22176p294**	352	0.006	0.006	-0.003	100	0.510	0.466	-0.100		species/Jp				0.348		Protein of unknown function (DUF1230)
**Jp_c22804p650**					98	0.429	0.427	-0.009		Jp						aldose 1-epimerase/galactose mutartotase-like superfamily protein
**Jp_c23049p629**	367	0.379	0.393	0.036	100	0.290	0.351	0.168		all				0.183		papain family cysteine protease/cathepsin propeptide inhibitor domain
**Jp_c23259p1067**	368	0.016	0.016	-0.008	100	0.400	0.396	-0.015		species/Jp				0.05		SIT4 phosphatase-associated family protein
**Jp_c23409p668**	368	0.024	0.024	-0.012	100	0.560	0.499	-0.127		all		2		0.205	Positive	glyoxalase/bleomycin resistance protein/dioxygenase superfamily
**Jp_c23680p1524**	368	0.014	0.019	0.279	100	0.390	0.484	0.191		all		1		0.115		copper amine oxidase family protein
**Jp_c23867p705**	367	0.003	0.003	-0.001	100	0.400	0.387	-0.040		species/Jp				0.374		peroxin 11D
**Jp_c23884p704**	368	0.011	0.011	-0.005	100	0.540	0.503	-0.080		species/Jp				0.572		signal peptide peptidase like-2
**Jp_c24821p611**	368	0.027	0.027	-0.014	100	0.320	0.284	-0.134		all	1	2		0.494	Positive	nodulin MtN3 family protein
**Jp_c25025p252**	339	0.024	0.035	0.321	100	0.470	0.496	0.048		all				0.341		ubiquitin-like superfamily/UBL5
**Jp_c25075p377**	368	0.416	0.419	0.005	100	0.560	0.451	-0.248		all	2			0	Negative	phenylcoumaran benzylic ether reductase/NmrA-like negative transcriptional regulator
**Jp_c25220p628**	368	0.033	0.032	-0.017	100	0.470	0.480	0.017		all				0.168		ribosomal protein L19e
**Jp_c25513p539**	359	0.435	0.456	0.046	100	0.450	0.476	0.050	LP06	all			1	0.033	Negative	ribosomal protein L6
**Jp_c26007p766**	368	0.003	0.003	-0.001	100	0.280	0.242	-0.163		species/Jp				0.102		glycine cleavage T-protein/aminomethyltransferase folate binding domain
**Jp_c26456p564**	368	0.171	0.170	-0.007						Lp						40S ribosomal protein S4; Provisional
**Jp_c26537p1091**	368	0.068	0.076	0.103	100	0.320	0.322	0.000		all		1		0.133		homeobox domain/Homeobox-leucine zipper protein family
**Jp_c26967p1321**	368	0.008	0.008	-0.004	100	0.530	0.500	-0.065		species/Jp			1	0.06	Negative	S-adenosylmethionine-dependent methyltransferase superfamily protein
**Jp_c27011p1002**	368	0.005	0.005	-0.003	100	0.530	0.494	-0.078		species/Jp				0.865		targeting protein for Xklp2 (TPX2)
**Jp_c27188p1706**	368	0.003	0.003	-0.001	99	0.505	0.475	-0.068		species/Jp				0.32		suppressor of abi3-5
**Jp_c27585p643**	367	0.409	0.455	0.100	100	0.460	0.502	0.080		all	1			0	Negative	alpha-L-arabinofuranosidase/kelch motif
**Jp_c27768p690**	368	0.285	0.271	-0.053	100	0.460	0.501	0.077		all				0.079		papain family cysteine protease/cathepsin propeptide inhibitor domain
**Jp_c27995p367**	363	0.008	0.014	0.396	100	0.540	0.499	-0.087		species/Jp				0.05		transcribed locus/BES1-interacting Myc-like protein 2
**Jp_c28163p1406**	368	0.022	0.027	0.189	100	0.160	0.148	-0.087		all		1		0.019		CCT motif family protein
**Jp_c28414p495**	303	0.251	0.398	0.369	97	0.423	0.489	0.131		all				0		cytochrome c oxidase;electron carriers
**Jp_c28904p670**	368	0.011	0.011	-0.005	100	0.350	0.488	0.279		species/Jp			2	0	Positive	ubiquitin-conjugating enzyme 28//E3 interaction residues
**Jp_c28943p445**	362	0.011	0.011	-0.006	100	0.380	0.387	0.012		species/Jp				0.543		ubiquitin-conjugating enzyme/ IQ-domain 26
**Jp_c28993p1003**	368	0.459	0.475	0.032	100	0.440	0.405	-0.091	JPL1	all				0.452		GRAS family transcription factor
**Jp_c28993p897**	367	0.019	0.019	-0.010	100	0.440	0.414	-0.068	JPL1	all	1	1		0.224	Positive	GRAS family transcription factor
**Jp_c29875p506**	368	0.003	0.003	-0.001	100	0.420	0.437	0.035		species/Jp				0.992		transcribed locus
**Jp_c30646p698**	363	0.003	0.003	-0.001	100	0.300	0.284	-0.063		species/Jp				0.135		mitochondrial carrier family protein (isoform 1)
**Jp_c31376p1552**	368	0.043	0.043	-0.022	100	0.560	0.497	-0.131		all				0.445		ATP binding;ATP-dependent helicases;DNA helicases
**Jp_c31636p544**	367	0.281	0.287	0.019	100	0.420	0.405	-0.042		all				0.477		methylenetetrahydrofolate reductase
**Jp_c32144p353**					99	0.535	0.489	-0.099		Jp						Protein kinase superfamily protein
**Jp_c32861p648**					95	0.326	0.414	0.207		Jp						ubiquitin-related modifier
**Jp_c33062p504**	362	0.008	0.008	-0.004	100	0.400	0.451	0.109		species/Jp				0.963		cytochrome c oxidase related
**Jp_c33476p566**	368	0.087	0.088	0.013	99	0.485	0.470	-0.036		all				0.897		CLP protease regulatory subunit
**Jp_c34152p781**	347	0.127	0.124	-0.024	99	0.515	0.502	-0.031		all			2	0.018	Positive	ribosomal protein L27
**Jp_c34224p545**	368	0.011	0.011	-0.005	100	0.450	0.418	-0.082		species/Jp				0.434		transcribed locus
**Jp_c34823p887**					95	0.516	0.495	-0.048		Jp						ribosomal S3A

Locus	Lodgepole pine				Jack pine				Linkage group	Data	Outlier Detection			Introgress	Direction	Annotation
	N	Ho	UHe	F	N	Ho	UHe	F			Species	Lodge	Jack			
**Jp_c34902p455**	365	0.005	0.005	-0.003	100	0.490	0.488	-0.009		species/Jp	1			0.768	Positive	copper/zinc superoxide dismutase
**Jp_c38919p628**	362	0.304	0.288	-0.057						Lp						caleosin-related family protein/responsive to dessication 20
**Jp_c40483p485**	368	0.005	0.005	-0.003	100	0.430	0.410	-0.055	JPL3	species/Jp	1			0.57	Positive	pyridine nucleotide-disulphide oxidoreductase
**Jp_c40483p739**	368	0.008	0.008	-0.004	100	0.430	0.410	-0.055	JPL3	species/Jp	1			0.624	Positive	pyridine nucleotide-disulphide oxidoreductase
**Jp_c40635p369**	368	0.000	0.000		100	0.510	0.480	-0.067		species/Jp				0.931		photosystem II subunit Q-2/Oxygen evolving enhancer protein 3
**Jp_c43016p279**	352	0.014	0.014	-0.007	100	0.340	0.309	-0.105		species/Jp				0.02		transcribed locus
**Jp_c43058p705**	366	0.011	0.011	-0.005	100	0.380	0.405	0.058		species/Jp	1			0.001	Positive	HSP20-like chaperones superfamily protein
**Jp_c43213p458**	368	0.019	0.019	-0.010	100	0.900	0.499	-0.812	LP07	all		2		0	Positive	lipid transfer protein/seed storage 25 albumin superfamily protein
**Jp_c43596p385**	367	0.005	0.005	-0.003	100	0.510	0.471	-0.088	JP13	species/Jp				0.106		peptide transporter
**Jp_c43942p268**	367	0.213	0.232	0.081	100	0.330	0.410	0.190		all				0.837		alpha/beta hydrolases superfamily/dienelactone hydrolase family
**Jp_c43969p509**	366	0.123	0.116	-0.066	99	0.525	0.501	-0.053		all				0.883		ribosomal protein L13e/breat basic conserved 1
**Jp_c44066p1378**	368	0.019	0.019	-0.010	100	0.410	0.401	-0.028		all				0.7		little nuclei like
**Jp_c44467p708**	368	0.005	0.005	-0.003	100	0.380	0.414	0.077		species/Jp				0.207		ubiquitin-conjugating enzyme
**Jp_c44595p1466**	363	0.146	0.181	0.193	100	0.460	0.414	-0.117		all				0.079		alpha-galactosidase 2/glycosyl hydrolase family 31
**Jp_c44637p536**	368	0.022	0.022	-0.011	100	0.470	0.502	0.059	LP07	all		2		0.579	Positive	coatomer alpha subunit/glycine-rich protein
**Jp_c44933p494**	368	0.024	0.024	-0.012	100	0.390	0.410	0.043		all	1	2		0.139	Positive	photosystem II reaction center protein
**Jp_c45253p360**					99	0.354	0.364	0.024		Jp						ribosomal L22e protein family
**Jp_c45986p341**	360	0.008	0.008	-0.004	100	0.450	0.496	0.089		species/Jp				0.089		ribosomal protein S14p/S29e family protein
**Jp_c46035p392**					100	0.390	0.410	0.043		Jp						ribosomal protein S13/S18 family
**Jp_c46992p573**	367	0.016	0.016	-0.008	100	0.370	0.372	0.000		species/Jp				0.21		zinc finer (C2H2 type) family protein
**JpLpc00337p904**	367	0.311	0.302	-0.029	100	0.040	0.039	-0.020	LP08	all			1	0.207		basic helix-loop-helix (bHLH) DNA-binding superfamily protein
**JpLpc00745p1003**	368	0.457	0.473	0.034	100	0.030	0.030	-0.015	LP09	all	removed			0.326		protein kinases/ubiquitin-protein ligase
**JpLpc00988p408**	368	0.011	0.011	-0.005	100	0.460	0.463	0.002		species/Jp				0.252		WRKY DNA binding domain
**JpLpc01929p219**	368	0.495	0.501	0.011	100	0.210	0.205	-0.032	JP09	all				0.001		CHASE domain containing histidine kinase protein
**JpLpc02715p437**	367	0.112	0.110	-0.013	99	0.515	0.495	-0.046		all				0		transcribed locus
**JpLpc02733p310**	368	0.011	0.011	-0.005	100	0.270	0.339	0.200		species/Jp	1			0.998	Positive	transcribed locus
**JpLpc03397p578**	359	0.206	0.203	-0.019	100	0.030	0.030	-0.015		all				0.33		SNARE associated golgi protein
**JpLpc03587p814**	367	0.395	0.375	-0.056	100	0.010	0.010	-0.005		species/Lp				0.615		protein kinase/casein kinase 1
**JpLpc04112p1237**	365	0.008	0.014	0.396	100	0.490	0.494	0.003	JP01	species/Jp				0.01		SSXT family protein
**JpLpc04112p131**	368	0.046	0.045	-0.024	100	0.010	0.010	-0.005		species/Lp	1			0.123	Positive	SSXT family protein
**JpLpc04689p1306**	325	0.403	0.405	0.004					LPL2	Lp						similar to Arabisopsis thaliana At1g70160/
**JpLpc04689p1619**	364	0.448	0.438	-0.024	100	0.000	0.000		LPL2	species/Lp				0.105		similar to Arabisopsis thaliana At1g70160/
**JpLpc05265p1240**	368	0.024	0.029	0.169	100	0.000	0.000			species/Lp				0.073		transcribed locus
**JpLpc06142p940**	368	0.497	0.488	-0.021	100	0.450	0.476	0.050		all	1			0.782	Negative	transcribed locus
**JpLpc07372p371**	368	0.484	0.497	0.025	100	0.040	0.039	-0.020		all				0.726		stabilizer of ion transporter SufD/polynucelotidyl transferase
**JpLpc07589p2036**	367	0.278	0.306	0.090	100	0.280	0.284	0.008		all	1			0.283	Negative	NADH-ubiquinone dehydrogenase/molybdopterin oxidoreductase
**JpLpc08428p772**	368	0.011	0.011	-0.005	100	0.530	0.448	-0.190		species/Jp				0.012		alpha and gamma adaptin binding protein
**JpLpc09880p1739**	362	0.086	0.082	-0.045	99	0.172	0.206	0.164		all				0		methyltransferase domain/tellurite resistance protein
**JpLpc10450p806**	368	0.440	0.434	-0.015	100	0.050	0.049	-0.026		all				0.166		myb-like DNA-binding domain
**JpLpc11477p234**	368	0.098	0.093	-0.051	99	0.000	0.000			species/Lp				0.075		Tetratricopeptide repeat (TRP)/FKBP-type peptidyl-prolyl cis-trans isomerase
**JpLpc15608p1838**	337	0.151	0.140	-0.082						Lp		3			Positive	Transducin family protein / WD-40 repeat family protein
**JpLpc16915p385**	368	0.111	0.105	-0.059	99	0.182	0.166	-0.100		all				0.056		transcribed locus
**JpLpc18130p528**	368	0.171	0.175	0.018	99	0.152	0.158	0.035		all				0.26		similar to Arabisopsis thaliana thaliana At5g13030

Locus	Lodgepole pine				Jack pine				Linkage group	Data	Outlier Detection			Introgress	Direction	Annotation
	N	Ho	UHe	F	N	Ho	UHe	F			Species	Lodge	Jack			
**JpLpc21364p981**	368	0.052	0.056	0.069	100	0.140	0.131	-0.075		all	1	3		0	Positive	myb-like DNA binding domain
**JpLpc21779p551**	368	0.079	0.076	-0.041	100	0.040	0.039	-0.020		all	1			0.377	Positive	Rab5-interacting family protein
**JpLpc23919p3213**	367	0.384	0.424	0.092	100	0.030	0.030	-0.015		all				0.811		response regulator 2
**JpLpc24416p627**	368	0.008	0.008	-0.004	100	0.460	0.474	0.024		species/Jp				0		HOPW1-1 interacting 1/aminotransferase class III
**JpLpc24944p305**	368	0.008	0.008	-0.004	99	0.535	0.473	-0.138		species/Jp				0.011		transcribed locus
**JpLpc26435p293**	368	0.484	0.501	0.032	100	0.060	0.058	-0.031		all				0.26		TATA-binding protein/cullin-associated and neddylation dissociated/HEAT repeats
**JpLpc30315p463**	367	0.305	0.299	-0.022	100	0.000	0.000			species/Lp				0.137		basic pentacysteine 6/GAGA binding protein-like family
**JpLpc30743p1175**	368	0.168	0.163	-0.032	100	0.370	0.382	0.026		all				0.563		thylakoid lumenal P17.1 protein
**JpLpc30808p665**	368	0.011	0.011	-0.005	97	0.371	0.341	-0.094		species/Jp	1			0.44	Positive	BTB/POZ domain and MATH domain 2
**JpLpc31632p126**	363	0.325	0.347	0.062	100	0.320	0.284	-0.134		all				0.31		cystatin domain
**JpLpc31862p774**	367	0.278	0.285	0.023	100	0.010	0.010	-0.005		species/Lp				0.432		chalcone-flavanone isomerase
**JpLpc31990p707**	368	0.019	0.019	-0.010	99	0.343	0.416	0.171	JPL2	all		1		0.024		homeobox 1
**JpLpc31990p90**	368	0.027	0.027	-0.014	100	0.460	0.495	0.067	JPL2	all		1		0.021		homeobox 1
**JpLpc32323p740**	367	0.022	0.027	0.189	100	0.240	0.227	-0.061		all	1		1	0.032	Positive	Aluminum induced protein with YGL and LRDR motifs
**JpLpc32835p1047**	368	0.457	0.475	0.037	100	0.060	0.058	-0.031		all				0.337		transcribed locus
**JpLpc33893p239**	368	0.016	0.016	-0.008	100	0.070	0.104	0.327		species/Jp	3			0.585	Positive	auxin-F-box protein 5
**JpLpc34244p1745**	365	0.890	0.495	-0.800	98	0.459	0.431	-0.071		all	1			0	Negative	Basic-leucine zipper transcription factor family protein
**JpLpc34265p968**	364	0.327	0.306	-0.070	100	0.110	0.104	-0.058		all				0		F-box like family protein
**JpLpc34784p179**	367	0.044	0.043	-0.022	99	0.455	0.456	-0.001		all				0.995		calreticulin family
**JpLpc35530p946**	358	0.310	0.313	0.009	99	0.182	0.183	-0.001		all				0.082		ethelene response factor
**JpLpc36107p918**	366	0.268	0.267	-0.004	100	0.180	0.165	-0.099		all				0.292		transcribed locus
**JpLpc36252p1327**	368	0.082	0.078	-0.042	100	0.020	0.020	-0.010		all	1			0.002	Positive	histone chaperone/global transription factor C
**JpLpc36728p680**	366	0.115	0.108	-0.061	100	0.560	0.490	-0.149		all				0		anitcondon-binding domain/RNA-processing
**JpLpc37132p434**	336	0.467	0.464	-0.008	100	0.000	0.000			species/Lp				0		
**JpLpc37447p1264**	368	0.011	0.011	-0.005	100	0.530	0.484	-0.100		species/Jp				0.082		Fcf2 pre-rRNA processing protein
**JpLpc38316p430**	305	0.148	0.142	-0.037						Lp						sucrose-6F-phosphate synthase
**JpLpc38375p1504**	367	0.439	0.471	0.066	100	0.100	0.095	-0.053	LP10/JP10	all				0.04		homeodomain-like superfamily
**JpLpc39429p214**	368	0.163	0.177	0.076	99	0.071	0.069	-0.037		all				0.23		SIN3-like 4
**JpLpc39841p549**	367	0.401	0.366	-0.094	99	0.374	0.384	0.023		all				0.034		similar to Arabisopsis thaliana At4g09830
**JpLpc39929p732**	368	0.087	0.083	-0.045	100	0.490	0.448	-0.100		all				0.109		SSXT family protein/translation elongation factor EF1A
**JpLpc39993p867**	368	0.033	0.037	0.126	100	0.210	0.189	-0.117		all		1		0.062		uncharacterized conserved protein (DUF2358)/SnoaL-like domain
**JpLpc39995p1987**	368	0.030	0.029	-0.015	100	0.520	0.486	-0.075	LP04/JP11	all				0		Telomerase activating protein Est1
**JpLpc40356p1400**	367	0.302	0.287	-0.057	97	0.031	0.031	-0.016		all	1			0	Negative	inorganic H+ pyrophosphatase
**JpLpc40793p1901**	368	0.014	0.014	-0.007	100	0.310	0.303	-0.028		species/Jp	1			0.062	Positive	Rtr1/RPAP2 family
**JpLpc41024p554**	368	0.071	0.068	-0.037						Lp						F-box family
**JpLpc41037p1156**	368	0.378	0.374	-0.011	100	0.010	0.010	-0.005		species/Lp				0.577		TMPIT-like protein
**JpLpc41319p340**	368	0.027	0.032	0.153	100	0.030	0.049	0.385		all	2	1		0.992	Positive	uncharacterized BCR, YbaB family COG0718
**JpLpc42885p286**	368	0.563	0.498	-0.130	100	0.300	0.356	0.153		all				0.158		DDRGK domain
**JpLpc44209p446**	368	0.073	0.071	-0.038	100	0.290	0.263	-0.107	LP11/JP08	all	removed			0.22		auxin response factor 1
**JpLpc44494p452**	366	0.019	0.019	-0.010	100	0.560	0.502	-0.122		all		1		0.053		transcribed locus
**JpLpc44713p1218**					98	0.531	0.442	-0.206		Jp			1		Negative	SEUSS-like 2
**JpLpc44713p1452**	368	0.410	0.429	0.043	100	0.120	0.131	0.078	LP07	all				0.016		SEUSS-like 2
**JpLpc44782p470**	368	0.022	0.022	-0.011	100	0.320	0.297	-0.084		all	1	2		0.335	Positive	KNOX1/2 domain/KNOTTED-like

Locus	Lodgepole pine				Jack pine				Linkage group	Data	Outlier Detection			Introgress	Direction	Annotation
	N	Ho	UHe	F	N	Ho	UHe	F			Species	Lodge	Jack			
**JpLpc44961p330**	368	0.429	0.426	-0.009	99	0.525	0.494	-0.069		all				0.735		transcribed locus
**JpLpc45225p571**	368	0.043	0.043	-0.022	99	0.202	0.183	-0.112	LP07	all	1	3		0.002	Positive	B-cell receptor-associated protein 31-like
**JpLpc45900p1071**	368	0.476	0.473	-0.007	95	0.032	0.031	-0.016		all				0.527		AP2 domain/integrase-type DNA-binding/eukaryotic translation initiation factor
**JpLpc47089p1628**	368	0.486	0.445	-0.095	100	0.050	0.049	-0.026		all				0.422		Dof-type zinc finger DNA-binding family protein
**JpLpc47089p1831**	366	0.016	0.016	-0.008	100	0.190	0.173	-0.105		species/Jp	2		1	0.249	Positive	Dof-type zinc finger DNA-binding family protein
**JpLpc47519p1846**	367	0.008	0.008	-0.004	100	0.200	0.212	0.053		species/Jp				0.259		ankyrin repeat family protein with DHHC zinc finger domain
**JpLpc47519p2237**	365	0.474	0.462	-0.028	99	0.131	0.123	-0.070		all				0.022		ankyrin repeat family protein with DHHC zinc finger domain
**JpLpc47778p1036**					100	0.030	0.030	-0.015		Jp						chlorophyll A-B binding family protein
**JpLpc48195p543**	366	0.505	0.501	-0.011	100	0.060	0.058	-0.031		all				0.342		NAD(P)-binding Rossmann-fold superfamily protein/short chain dehydrogenase
**JpLpc48449p524**					100	0.280	0.270	-0.042		Jp						transcribed locus
**JpLpc49274p383**	367	0.267	0.259	-0.033	100	0.000	0.000			species/Lp				0.015		double-stranded DNA binding
**JpLpc49873p886**	368	0.003	0.003	-0.001	100	0.480	0.499	0.034	JP11	species/Jp				0.234		protein of unknown function (DUF1118)
**JpLpc50195p453**	291	0.113	0.119	0.048						Lp						complex I subunit
**JpLpc50715p1991**	368	0.033	0.032	-0.017	100	0.470	0.494	0.044		all				0.012		ubiquitin-like superfamily
**JpLpc50903p425**	368	0.005	0.005	-0.003	100	0.390	0.434	0.096		species/Jp				0.917		transcribed locus
**JpLpc54166p882**	368	0.304	0.315	0.033	100	0.020	0.020	-0.010		all				0.123		
**JpLpc54814p220**	368	0.247	0.268	0.075	100	0.020	0.020	-0.010	LPL3	all				0		basic helix-loop-helix (bHLH) DNA-binding superfamily protein
**JpLpc54814p513**	367	0.300	0.316	0.050	100	0.050	0.049	-0.026	LPL3	all			1	0.114		basic helix-loop-helix (bHLH) DNA-binding superfamily protein
**JpLpc56344p652**	368	0.451	0.444	-0.018	100	0.150	0.139	-0.081		all				0.026		cell elongation protein/DWARF 1/calmodulin binding
**JpLpc57540p66**	366	0.508	0.439	-0.158	100	0.240	0.297	0.187		all				0		RNA binding motif/dicer-like 1
**JpLpc60957p494**	368	0.470	0.473	0.004	100	0.110	0.104	-0.058		all				0		cysteine proteinases superfamily protein
**JpLpc61376p1097**	368	0.386	0.408	0.054	100	0.010	0.010	-0.005		species/Lp				0.259		protein kinase/mitogen-activated protein kinase
**JpLpc63491p915**	368	0.019	0.019	-0.010	100	0.240	0.256	0.059		all	2	1		0.002	Positive	cytokinin response factor/AP2 domain
**JpLpc65526p1833**	368	0.473	0.490	0.034	100	0.040	0.039	-0.020		all				0.108		AP2 domain/integrase-type DNA-binding superfamily
**JpLpc66545p1207**	368	0.019	0.019	-0.010	99	0.081	0.078	-0.042		all	2		1	0.02	Positive	transcribed locus
**JpLpc66974p221**	365	0.438	0.453	0.030	100	0.550	0.448	-0.234		all	1			0.048	Negative	enhanced downy mildew 2 (EDM2)
**JpLpc68435p382**	367	0.510	0.494	-0.033	100	0.050	0.049	-0.026		all				0.661		Acyl-CoA N-acyltransferases superfamily protein
**JpLpc71773p809**	365	0.471	0.500	0.057	99	0.465	0.440	-0.062		all				0.166		ribosomal protein L18ae family
**JpLpc72416p369**	364	0.349	0.377	0.073	100	0.020	0.020	-0.010		all				0.037		ARF-GAP domain 8/putative GTPase activating protein for Arf
**JpLpc72792p1173**	367	0.033	0.048	0.317					LP12	Lp		1				polyketide cyclase/dehydrase and lipid transport superfamily/START_STARD2_7 like
**JpLpc73084p710**					100	0.360	0.345	-0.049		Jp						phytochrome-associated protein phosphatase type 2C
**JpLpc74417p752**	367	0.534	0.497	-0.077						Lp						Protein of unknown function (DUF2488)
**JpLpc74576p546**	368	0.003	0.003	-0.001	100	0.220	0.227	0.027		species/Jp				0.309		transcribed locus
**JpLpc75275p653**	368	0.098	0.113	0.130	100	0.530	0.401	-0.329		all				0		transcribed locus
**JpLpc75741p1236**	368	0.489	0.486	-0.009	100	0.100	0.095	-0.053		all				0.229		kinase related protein of unknown functin
**JpLpc79147p1879**	368	0.063	0.061	-0.032	100	0.090	0.104	0.134		all	1	1		0.45	Positive	transcribed locus
**JpLpc79197p531**	368	0.497	0.493	-0.011	100	0.070	0.068	-0.036	LP10/JP1C	all			1	0.286		homolog of anti-oxidant 1/heavy-metal associated domain/copper chaperone
**JpLpc79522p772**					83	0.434	0.414	-0.055		Jp						transcribed locus
**JpLpc79566p1158**	368	0.514	0.500	-0.029	100	0.200	0.181	-0.111		all				0.164		transcribed locus
**JpLpc80697p862**	368	0.465	0.501	0.070	100	0.400	0.345	-0.166		all				0.002		AWPM-19 family (membrane)/ peptidoglycan-binding LysM domain-containing
**JpLpc82733p437**	365	0.496	0.499	0.004	100	0.020	0.020	-0.010		all				0.031		basic transcription factor 3/NAC domain
**JpLpc82733p81**	366	0.005	0.005	-0.003	100	0.550	0.502	-0.101		species/Jp				0.015		basic transcription factor 3/NAC domain
**JpLpc85542p1333**					94	0.468	0.458	-0.027		Jp						geranylgeranyl pyrophosphate synthase 1

Locus	Lodgepole pine				Jack pine				Linkage group	Data	Outlier Detection			Introgress	Direction	Annotation
	N	Ho	UHe	F	N	Ho	UHe	F			Species	Lodge	Jack			
**JpLpc86157p398**	367	0.161	0.166	0.031	100	0.090	0.086	-0.047	JPLP2	all	1		1	0.169	Positive	RNA recognition motif/SC35-like splicing factor 28
**JpLpc86157p76**	340	0.179	0.178	-0.010	99	0.091	0.087	-0.048	JPLP2	all	1		1	0.289	Positive	RNA recognition motif/SC35-like splicing factor 28
**Lodgc103p389**	368	0.467	0.426	-0.098	100	0.210	0.220	0.040		all				0.039		4-hydroxy-3-methylbut-2-enyl diphosphate reductase
**Lodgc1040p150**	368	0.117	0.129	0.094	100	0.010	0.010	-0.005		species/Lp				0.221		ADR1-like/leucine rich repeat/NB-ARC domain
**Lodgc1065p551**	368	0.052	0.050	-0.026						Lp						ubiquitin-specific protease 13
**Lodgc1073p453**	367	0.360	0.371	0.028	100	0.010	0.010	-0.005		species/Lp				0.402		glutamyl-tRNA reductase family protein
**Lodgc1087p211**	368	0.239	0.270	0.112	100	0.000	0.000		LP13	species/Lp	1	3		0.005	Positive	proteasome alpha type 7
**Lodgc1092p492**	363	0.267	0.267	-0.002	100	0.010	0.010	-0.005		species/Lp				0.068		zinc-binding dehydrogenase/oxidoreductase
**Lodgc1110p421**	368	0.408	0.426	0.042	100	0.050	0.049	-0.026		all				0.002		NAC domain containing protein 71
**Lodgc1141p278**	363	0.215	0.254	0.152	100	0.000	0.000			species/Lp				0.042		DnaJ homolog 3
**Lodgc118p350**	365	0.427	0.467	0.083	100	0.010	0.010	-0.005	LP13	species/Lp	2	3		0	Positive	sedoheptulose-bisphosphate/fructose-1-6-bisphosphate - chloroplast like
**Lodgc1193p223**	365	0.323	0.304	-0.066	100	0.010	0.010	-0.005		species/Lp				0.069		transcribed locus
**Lodgc1214p281**	366	0.363	0.337	-0.078	100	0.000	0.000			species/Lp				0.239		delta subunit of Mt ATP synthase
**Lodgc123p319**	368	0.337	0.331	-0.018	100	0.010	0.010	-0.005		species/Lp				0.983		LSD1 like zinc finger
**Lodgc1249p448**	302	0.487	0.412	-0.183						Lp						pyruvate phosphate dikinase/pyruvate binding domain
**Lodgc1316p295**	362	0.276	0.291	0.051						Lp						adolase superfamily protein/fructose-bisphosphate adolase class I
**Lodgc1407p248**	324	0.043	0.111	0.609	100	0.000	0.000			species/Lp				0		stress-inducible protein, putative/sialyltransferase family protein
**Lodgc1490p507**	368	0.057	0.056	-0.029						Lp						ubiquinal cytochrome reductase transmembrane region/iron-sulfur subunit
**Lodgc1496p263**	368	0.144	0.148	0.023	100	0.000	0.000			species/Lp				0.369		beta-ketoacyl reductase/KR domain/short chain dehydrogenase
**Lodgc1562p404**	368	0.486	0.493	0.011	100	0.030	0.030	-0.015		all				0.63		fibrillin family protein/Plastid-lipid associated protein PAP
**Lodgc157p477**	352	0.134	0.154	0.131	100	0.020	0.020	-0.010		all				0.01		adenosylmethionine decarboxylase
**Lodgc15p411**	366	0.369	0.370	0.002	100	0.260	0.270	0.033		all				0.009		YGGT family protein
**Lodgc1623p352**	365	0.299	0.302	0.009						Lp						B-box type zinc finger protein with CCT domain
**Lodgc1794p389**	360	0.422	0.431	0.020	100	0.450	0.392	-0.155		all				0.177		4-coumarate-CoA ligase enzyme (PT4CL1)
**Lodgc1891p418**	368	0.283	0.284	0.004	100	0.580	0.482	-0.208		all				0.04		transcribed locus
**Lodgc189p276**	367	0.267	0.244	-0.098	100	0.010	0.010	-0.005		species/Lp				0.083		ATP-citrate synthase
**Lodgc1909p373**	365	0.255	0.277	0.079	100	0.110	0.104	-0.058		all				0.519		beta-ketoacyl synthase, N-terminal domain/fatty acid biosynthesis 1
**Lodgc1931p524**	368	0.277	0.277	-0.002	100	0.020	0.020	-0.010		all				0.021		transcribed locus
**Lodgc2047p463**	368	0.546	0.494	-0.108	100	0.380	0.333	-0.145		all				0.058		helix-loop-helix DNA binding domain
**Lodgc2049p390**	368	0.215	0.221	0.028	100	0.010	0.010	-0.005		species/Lp				0.634		RING/U-box superfamily protein
**Lodgc218p506**	367	0.112	0.115	0.029	100	0.000	0.000			species/Lp		1		0.126	Negative	transcribed locus
**Lodgc2226p408**	366	0.372	0.354	-0.051	100	0.330	0.328	-0.012		all				0.334		IQ calmodulin-binding motif
**Lodgc2304p514**	367	0.240	0.274	0.123	100	0.010	0.010	-0.005	LP14	species/Lp	1	3		0.24	Positive	triosephosphate isomerase
**Lodgc2347p440**	358	0.263	0.233	-0.131	98	0.429	0.492	0.125	JP12	all				0.497		serine-rich protein-related
**Lodgc2413p445**	368	0.111	0.120	0.068	99	0.000	0.000			species/Lp		1		0.02		histone deacetylase
**Lodgc2449p408**	366	0.189	0.197	0.042	100	0.110	0.104	-0.058		all				0.118		membrane-associated mannitol-induced
**Lodgc2458p469**					100	0.280	0.270	-0.042		Jp						CLP protease
**Lodgc2485p527**	367	0.428	0.465	0.079	100	0.440	0.469	0.056		all				0.705		transcribed locus
**Lodgc2532p1120**	368	0.492	0.489	-0.006	100	0.050	0.049	-0.026		all				0.081		Ras family/miro-like protein/signal recognition particle receptor beta subunit
**Lodgc2606p320**	367	0.180	0.182	0.008	100	0.190	0.173	-0.105	JP13	all				0.003		ADP-ribosylation factor family/G protein alpha subunit
**Lodgc2627p673**	367	0.471	0.497	0.050	100	0.080	0.077	-0.042		all				0.357		2-oxoglutarate (2OG) and Fe(II) dependent oxygenase superfamily protein
**Lodgc2649p345**	368	0.117	0.120	0.023	100	0.010	0.010	-0.005		species/Lp				0.388		thioredoxin
**Lodgc2743p497**	359	0.223	0.244	0.086	100	0.030	0.030	-0.015		all				0.007		eIF4-gamma/ARM repeat superfamily protein

Locus	Lodgepole pine				Jack pine				Linkage group	Data	Outlier Detection			Introgress	Direction	Annotation
	N	Ho	UHe	F	N	Ho	UHe	F			Species	Lodge	Jack			
**Lodgc2792p662**	366	0.448	0.496	0.095	98	0.235	0.253	0.069		all				0.184		
**Lodgc2798p514**	368	0.486	0.467	-0.042	100	0.010	0.010	-0.005		species/Lp				0.193		rhamnose biosynthesis 1/polysaccharide biosynthesis/NAD dependent epimerase
**Lodgc2800p195**	367	0.158	0.146	-0.086	100	0.000	0.000			species/Lp				0.67		myb-like DNA-binding domain
**Lodgc2828p334**	367	0.365	0.353	-0.034	100	0.010	0.010	-0.005		species/Lp				0.177		polysaccharide biosynthesis/NAD dependent epimerase/dyhydroflavonol 4-reductase
**Lodgc3011p422**	314	0.643	0.499	-0.291						Lp						elongation factor
**Lodgc3248p281**					85	0.024	0.023	-0.012		Jp						protein kinase/tyrosine superfamily protein
**Lodgc3290p342**	356	0.292	0.292	-0.003	100	0.000	0.000			species/Lp				0.032		photosystem II reaction center PSB28 protein
**Lodgc3293p323**	364	0.503	0.481	-0.048	100	0.020	0.020	-0.010		all				0.654		Tetratricopeptide repeat (TRP) superfamily protein
**Lodgc3482p180**	366	0.527	0.485	-0.089	100	0.000	0.000			species/Lp				0.59		RAN GTPase 3/RNA helicase/miro-like protein/Ras family
**Lodgc348p360**	368	0.516	0.482	-0.074	99	0.040	0.040	-0.021		all				0.187		glutamate:glyoxylate aminotransferase
**Lodgc389p393**	367	0.319	0.337	0.052	99	0.030	0.030	-0.015		all				0.002		dehydrin
**Lodgc408p638**	367	0.267	0.251	-0.064	100	0.030	0.030	-0.015		all				0.006		IQ calmodulin-binding motif
**Lodgc4104p268**	358	0.849	0.501	-0.699	100	0.200	0.197	-0.021		all				0		purine biosynthesis 4
**Lodgc415p436**	367	0.256	0.278	0.076	100	0.000	0.000			species/Lp				0.072		ribosomal protein 1/L3
**Lodgc4293p449**	368	0.141	0.131	-0.076	100	0.000	0.000			species/Lp				0.341		trypsin family protein
**Lodgc4306p424**	366	0.467	0.475	0.015	100	0.280	0.309	0.090		all				0.012		nucleotide-diphospho-sugar transferases superfamily
**Lodgc4405p428**	368	0.212	0.219	0.031	100	0.010	0.010	-0.005		species/Lp				0.014		peroxin 14/peroxisomal membrane anchor protein
**Lodgc4407p669**	368	0.266	0.277	0.037	100	0.060	0.058	-0.031		all			1	0.042		PGRS-like
**Lodgc4455p208**	367	0.436	0.414	-0.055	99	0.455	0.501	0.088		all	1			0.318	Negative	Plant protein of unknown function (DUF868)
**Lodgc447p278**	367	0.447	0.356	-0.255					LP12	Lp						actin depolymerizing factor/tropomyosin-type actin-binding protein
**Lodgc4517p609**	334	0.368	0.369	0.002						Lp						protein tyrosine phosphatase
**Lodgc469p206**	357	0.353	0.372	0.050	99	0.000	0.000			species/Lp				0.002		cysteine proteinases/cathepsin propeptide inhibitor domain/papain cysteine protease
**Lodgc478p476**	367	0.153	0.164	0.068	100	0.320	0.333	0.036		all	1			0.012	Negative	alpha/beta hydrolase family
**Lodgc4922p383**	368	0.505	0.499	-0.014	100	0.090	0.104	0.134		all				0.588		2-oxoglutarate (2OG) and Fe(II) dependent oxygenase superfamily protein
**Lodgc5121p406**	367	0.376	0.402	0.063	100	0.020	0.020	-0.010		all				0.19		Protein of unknown function (DUF1218)
**Lodgc5297p86**	367	0.150	0.143	-0.046	100	0.330	0.466	0.288	JP12	all				0.649		RING 1A
**Lodgc5447p89**	368	0.288	0.288	-0.002	100	0.020	0.020	-0.010		all				0.983		Alba DNA/RNA binding protein
**Lodgc5453p480**	368	0.239	0.235	-0.018	100	0.270	0.290	0.065		all	2			0.118	Negative	nodulin-like/major facilitator superfamily protein
**Lodgc5567p169**	368	0.188	0.175	-0.076	100	0.000	0.000			species/Lp				0.085		photosystem I reaction centre (PSAN or PSI-N)
**Lodgc5620p213**	363	0.171	0.201	0.148						Lp						dormancy-auxin associated protein/heat shock protein
**Lodgc5646p131**	360	0.136	0.127	-0.073	91	0.066	0.085	0.216		all				0		ARM repeat superfamily protein
**Lodgc570p462**	368	0.101	0.105	0.044	100	0.000	0.000			species/Lp				0.765		similar to ewsr1b
**Lodgc576p498**	367	0.324	0.327	0.008	100	0.070	0.068	-0.036		all				0.329		cysteine-rich receptor like protein kinase/similar to copia protein (gag-int-pol protein)
**Lodgc6047p607**	366	0.139	0.135	-0.037	100	0.120	0.113	-0.064	LP06	all	1			0.672	Negative	ACD1-like
**Lodgc623p588**	368	0.266	0.315	0.154	100	0.010	0.010	-0.005		species/Lp				0.649		lactate/malate dehydrogenase, NAD binding domain
**Lodgc6246p531**	368	0.291	0.293	0.006	100	0.020	0.020	-0.010		all				0.671		peroxin 22
**Lodgc624p1141**	368	0.410	0.398	-0.033	100	0.020	0.020	-0.010		all				0.974		chloroplast sulphur E/Fe-S metabolism associated domain
**Lodgc642p886**	366	0.503	0.459	-0.098	100	0.010	0.010	-0.005		species/Lp				0.087		GATA type zinc finger transcription factor family protein
**Lodgc6501p370**	352	0.608	0.494	-0.232						Lp						Annexin
**Lodgc6505p421**	368	0.261	0.247	-0.058	100	0.430	0.401	-0.078		all				0.911		RING/U-box superfamily protein
**Lodgc654p579**	365	0.312	0.320	0.024	100	0.020	0.020	-0.010		all				0.66		cupin domain/1,2-dihydroxy-3-keto-5-methylthiopentene dioxygenase
**Lodgc6568p554**	368	0.329	0.366	0.100	100	0.100	0.131	0.232	JP09	all			2	0.01		flavanone 3-hydroxylase
**Lodgc6868p131**	368	0.367	0.354	-0.037	100	0.080	0.095	0.158		all				0.046		Drought-responsive family protein

Locus	Lodgepole pine				Jack pine				Linkage group	Data	Outlier Detection			Introgress	Direction	Annotation
	N	Ho	UHe	F	N	Ho	UHe	F			Species	Lodge	Jack			
**Lodgc7085p286**	366	0.385	0.378	-0.020	100	0.040	0.039	-0.020		all				0.15		Peptidase M76 family/Ku70-binding protein
**Lodgc7446p377**	365	0.523	0.491	-0.068	100	0.010	0.010	-0.005		species/Lp				0.674		flavin-binding monooxygenase family protein
**Lodgc758p388**	368	0.226	0.241	0.063	100	0.000	0.000			species/Lp				0.367		2-phosphoglycolate pohsphatase 1
**Lodgc7681p243**	361	0.363	0.489	0.256						Lp						cytochrome B5 isoform B
**Lodgc792p190**	368	0.207	0.215	0.038	100	0.020	0.020	-0.010		all				0.043		4-aminobutyrate transaminase/pyridoxal phosphate dependent transferases
**Lodgc889p498**	368	0.465	0.489	0.049	100	0.030	0.030	-0.015		all				0.001		UDP-glucose/GDP-mannose dehydrogenase family UDP binding domain
**Lodgc910p162**	336	0.420	0.380	-0.106	91	0.011	0.011	-0.006		species/Lp				0.191		glutamate decarboxylase/pyridoxal-dependent
**Lodgc914p496**	368	0.139	0.134	-0.037	100	0.000	0.000			species/Lp				0.862		tetraspanin family
**Lodgc978p318**	367	0.436	0.423	-0.033	100	0.550	0.491	-0.125	JP05	all				0.002		phosphoglycerate kinase
**Lp_c00150p459**	367	0.014	0.014	-0.007	100	0.130	0.173	0.244		species/Jp	2			0.353	Positive	circadian clock associated 1
**Lp_c00150p61**	368	0.351	0.345	-0.017	100	0.020	0.020	-0.010		all				0.011		circadian clock associated 1
**Lp_c00228p801**	362	0.533	0.495	-0.079	100	0.460	0.501	0.077		all				0.577		ribosomal protein L19 family
**Lp_c00318p734**	368	0.342	0.364	0.059	100	0.110	0.122	0.095		all				0.262		phosphofruktokinase family protein
**Lp_c00579p455**	362	0.434	0.473	0.082	100	0.030	0.030	-0.015		all				0.002		AUX/IAA family
**Lp_c00925p827**	367	0.253	0.238	-0.068	100	0.010	0.010	-0.005		species/Lp				0.546		calcineurin-like metallo-phosphoesterase superfamily protein
**Lp_c00983p1180**	367	0.354	0.376	0.057	100	0.520	0.499	-0.047		all	1			0.271	Negative	tetratricopeptide repeat like/basic-leucine zipper transcription factor
**Lp_c01140p845**	366	0.262	0.244	-0.076	100	0.000	0.000			species/Lp				0.133		tetratricopeptide repeat
**Lp_c01467p496**	368	0.147	0.150	0.020	100	0.120	0.113	-0.064		all	1			0	Negative	protein of unknown function (DUF538)
**Lp_c01502p915**	354	0.506	0.495	-0.022	94	0.340	0.360	0.051		all				0.003		calcineurin-like metallo-phosphoesterase superfamily protein
**Lp_c01813p1225**	367	0.240	0.228	-0.055	100	0.020	0.020	-0.010		all			1	0.299		biotin/lipoyl attachment domain-containing protein
**Lp_c02035p862**	368	0.416	0.425	0.021	100	0.050	0.049	-0.026		all				0.341		protein containing PDZ domain, K-box domain, and TPR region
**Lp_c02191p296**	368	0.492	0.498	0.012	100	0.030	0.030	-0.015	LPL4	all				0.917		transketolase
**Lp_c02191p997**	368	0.277	0.277	-0.002	100	0.010	0.010	-0.005	LPL4	species/Lp				0.71		transketolase
**Lp_c02598p1040**	316	0.481	0.464	-0.039						Lp						similar to Arabisopsis thaliana At1g62780
**Lp_c02648p549**	314	0.484	0.492	0.014						Lp						GHMP kinase family protein
**Lp_c02821p599**					98	0.459	0.459	-0.007		Jp						protein kinase superfamily protein
**Lp_c03449p321**	368	0.427	0.492	0.131	100	0.010	0.010	-0.005		species/Lp				0.214		B-Box type zinc-finger like protein
**Lp_c04005p661**	363	0.854	0.491	-0.742	99	0.010	0.010	-0.005		species/Lp				0		xyloglucan endo-transflycosylase
**Lp_c04005p977**	364	0.473	0.485	0.025	100	0.010	0.010	-0.005		species/Lp				0.005		xyloglucan endo-transflycosylase
**Lp_c04157p375**	368	0.239	0.227	-0.054	100	0.000	0.000			species/Lp				0.1		endoplasmic reticulum vesicle transporter
**Lp_c04318p2154**	368	0.207	0.215	0.038	100	0.000	0.000		LP14	species/Lp		3		0.267	Positive	carbohydrate binding molecule/starch branching enzyme 2.2
**Lp_c04466p3014**	346	0.486	0.459	-0.060						Lp						transducin/WD-40 repeat-like superfamily
**Lp_c04493p319**	366	0.462	0.494	0.064	100	0.040	0.058	0.313		all				0.087		magnesium transporter
**Lp_c05328p985**	368	0.375	0.381	0.014	100	0.190	0.189	-0.011		all				0.679		phosphogluconate dehydrogenase/glyoxylate reductase 1
**Lp_c07286p308**	367	0.433	0.406	-0.070	100	0.280	0.242	-0.163		all				0.115		chloroplast stem-loop binding protein/NAD dependent epimerase/dehydratase family
**Lp_c07630p326**	368	0.519	0.493	-0.054	100	0.330	0.316	-0.051		all				0.503		pectin lyase-like superfamily protein/glycosyl hydrolases family 28
**Lp_c07997p838**	365	0.515	0.484	-0.066	100	0.050	0.049	-0.026		all				0.27		cobalamin synthesis/plastid transcriptionaly active 17
**Lp_c08410p197**	367	0.455	0.466	0.023	100	0.040	0.039	-0.020		all			1	0.115		proteasome beta subunit
**Lp_c09201p186**	366	0.396	0.394	-0.007	99	0.020	0.020	-0.010	LPL5	all				0.988		ATP synthase delta-subunit
**Lp_c09201p641**	367	0.417	0.383	-0.090	100	0.030	0.030	-0.015	LPL5	all				0.346		ATP synthase delta-subunit
**Lp_c10619p774**	368	0.413	0.424	0.025	100	0.500	0.490	-0.026		all			1	0.043	Negative	dehydration responsive/ S-adenosyl-L-methionine-dependent methyltransferase
**Lp_c10940p79**	366	0.456	0.481	0.050	100	0.020	0.020	-0.010		all				0.372		Glycosyl transferase family 90
**Lp_c11275p339**	368	0.508	0.482	-0.056	100	0.020	0.020	-0.010		all				0.886		thioredoxin superfamily protein

Locus	Lodgepole pine				Jack pine				Linkage group	Data	Outlier Detection			Introgress	Direction	Annotation
	N	Ho	UHe	F	N	Ho	UHe	F			Species	Lodge	Jack			
**Lp_c12025p1415**	368	0.231	0.229	-0.009	100	0.030	0.030	-0.015		all				0.738		core-2/I-branching beta-1,6-N-acetylglucosaminyltransferase family protein
**Lp_c13667p869**	368	0.166	0.157	-0.059	100	0.010	0.010	-0.005		species/Lp				0.39		proteasome beta subunit/N-terminal nucleophile aminohydrolasses superfamily protein
**Lp_c13778p292**	347	0.369	0.480	0.230	100	0.470	0.488	0.032	JPLP3	all	1		1	0.001	Negative	purple acid phosphatase 18
**Lp_c13778p747**	367	0.411	0.442	0.067	100	0.300	0.396	0.239	JPLP3	all	1		1	0.145	Negative	purple acid phosphatase 18
**Lp_c14079p1373**	363	0.551	0.488	-0.130	99	0.455	0.394	-0.159		all	1			0.186	Negative	enolase
**Lp_c14699p1329**	368	0.046	0.045	-0.024						Lp						haloacid dehalogenase-like hydrolase superfamily protein
**Lp_c14699p706**	368	0.500	0.500	-0.001	100	0.130	0.122	-0.070		all			1	0.04		haloacid dehalogenase-like hydrolase superfamily protein
**Lp_c14947p720**	368	0.429	0.488	0.119	100	0.060	0.058	-0.031		all				0.019		ribosomal protein L1p/L10e family
**Lp_c15030p593**	304	0.368	0.379	0.026						Lp						senescence associated family protein
**Lp_c16370p922**	367	0.308	0.311	0.008	100	0.040	0.039	-0.020		all				0.989		MAP kinase kinase 6/protein tyrosine kinase
**Lp_c16943p1584**	368	0.109	0.108	-0.010	100	0.030	0.030	-0.015		all				0.005		3-ketoacyl-CoA synthase/chalcone and stilbene synthases/FAE1-Type III polyketide synthase
**Lp_c17356p659**	356	0.480	0.476	-0.010	97	0.258	0.270	0.040		all				0.001		myb-domain protein/DNA-binding
**Lp_c18494p368**	368	0.478	0.476	-0.006	100	0.050	0.049	-0.026		all				0.603		glucose-6-phosphate dehydrogenase 6
**Lp_c18615p1158**	366	0.194	0.193	-0.007	100	0.040	0.039	-0.020		all				0.749		ATP citrate lyase/succinyl-CoA sythetase beta-chain
**Lp_c19125p748**	352	0.040	0.039	-0.020	98	0.000	0.000			species/Lp				0		pyruvate kinase family protein
**Lp_c19571p508**	368	0.293	0.277	-0.061	100	0.010	0.010	-0.005		species/Lp				0.013		RNA 3'-terminal phosphate cyclase/enolpyruvate transferase, alpha/beta
**Lp_c19870p302**	368	0.486	0.498	0.022	100	0.080	0.077	-0.042		all				0.005		protease inhibitor/seed storage/LTP family
**Lp_c20503p566**	368	0.299	0.302	0.008	100	0.000	0.000		LP08	species/Lp				0.195		RAN binding domain protein
**Lp_c20831p1035**	356	0.140	0.145	0.031	99	0.020	0.020	-0.010		all				0		NIN like protein 7/PB1 domain
**Lp_c21093p679**	368	0.514	0.489	-0.051	100	0.010	0.010	-0.005		species/Lp				0.008		proteasome subunit alpha type 1
**Lp_c21261p452**	368	0.451	0.469	0.038						Lp						RNA-binding KH domain-containing protein
**Lp_c21366p1031**	363	0.375	0.425	0.118	100	0.010	0.010	-0.005		species/Lp				0.01		GDSL-like lipase acylhydrolase superfamily protein
**Lp_c21536p992**	367	0.226	0.230	0.014	100	0.440	0.437	-0.011		all				0		glycosyl hydrolase superfamily protein/endoxyloglucan transferase A3
**Lp_c21696p443**	368	0.440	0.404	-0.092	100	0.000	0.000			species/Lp				0.194		fatty acid hydroxylase superfamily
**Lp_c21731p403**	368	0.500	0.479	-0.045	100	0.080	0.077	-0.042		all				0.546		haloacid dehalogenase-like hydrolase superfamily protein
**Lp_c21879p397**	368	0.209	0.200	-0.046	100	0.090	0.086	-0.047		all				0.338		Acyl CoA binding protein
**Lp_c22300p474**	366	0.030	0.035	0.139						Lp						serine carbosypeptidase like
**Lp_c22393p548**	368	0.427	0.423	-0.010	100	0.010	0.010	-0.005	LP09	species/Lp				0.599		protein kinases/ubiquitin-protein ligase
**Lp_c22542p803**	368	0.429	0.430	0.001	100	0.410	0.410	-0.006		all				0.777		glutamate synthase (ferrodoxin)/NADH-dependent
**Lp_c22767p940**	360	0.197	0.196	-0.009	99	0.242	0.214	-0.138		all				0.133		arabinogalactan protein 2
**Lp_c23304p610**	368	0.421	0.439	0.040	99	0.455	0.443	-0.031		all				0		chaperonin-60 alpha/TCP-1
**Lp_c23425p1676**	311	0.354	0.342	-0.035						Lp						BEL1-like homeodomain/DNA binding transcription factor
**Lp_c23768p576**	368	0.473	0.492	0.038	100	0.430	0.494	0.125		all				0.001		casein kinase/protein tyrosine kinase
**Lp_c23779p298**	365	0.153	0.156	0.013						Lp						zinc-binding alcohol dehydrogenase, NAD binding/cinnamyl alcohol dehydrogenase 5
**Lp_c23779p894**	363	0.201	0.224	0.100						Lp						zinc-binding alcohol dehydrogenase, NAD binding/cinnamyl alcohol dehydrogenase 5
**Lp_c24197p836**	368	0.435	0.428	-0.017	100	0.030	0.030	-0.015		all				0.462		RED-like protein
**Lp_c24207p657**					95	0.063	0.100	0.367		Jp						chalcone-flavanone isomerase
**Lp_c24391p1138**	368	0.473	0.501	0.054	100	0.050	0.049	-0.026	JPLP4	all				0.305		ARF-GAP domain 6/putative GTPase activating protein for Arf
**Lp_c24391p444**	367	0.482	0.501	0.035	100	0.050	0.049	-0.026	JPLP4	all				0.464		ARF-GAP domain 6/putative GTPase activating protein for Arf
**Lp_c24502p363**	367	0.460	0.486	0.052	99	0.020	0.020	-0.010		all				0.001		modified of rudimentary protein (Mod (r))
**Lp_c24551p196**	367	0.057	0.056	-0.029						Lp						glycosyl hydrolase superfamily protein
**Lp_c24551p2210**	368	0.332	0.335	0.008	100	0.000	0.000			species/Lp				0.949		glycosyl hydrolase superfamily protein
**Lp_c24582p834**	368	0.448	0.463	0.030	100	0.000	0.000			species/Lp				0.059		ATPase family associated (AAA)/trisphosphate hydrolases superfamily

Locus	Lodgepole pine				Jack pine				Linkage	Data	Outlier Detection			Introgress	Direction	Annotation
	N	Ho	UHe	F	N	Ho	UHe	F	group		Species	Lodge	Jack			
**Lp_c24702p966**	363	0.471	0.482	0.021	100	0.030	0.030	-0.015		all		1		0.452	Positive	FAR-17a/AIG1-like protein
**Lp_c24728p554**	368	0.429	0.498	0.137	100	0.030	0.049	0.385		all				0.002		ribosomal protein S4 (RPS4A)
**Lp_c25137p1866**	368	0.514	0.499	-0.031	100	0.030	0.030	-0.015		all				0.933		COBRA-like extracellular glycosyl-phosphatidyl inositol-anchored protein family
**Lp_c25194p1160**	341	0.422	0.496	0.147	100	0.070	0.068	-0.036		all				0.274		Aluminum induced protein with YGL and LRDR motifs
**Lp_c25309p582**	368	0.424	0.432	0.018	100	0.030	0.030	-0.015		all				0.008		chaperone protein dnaJ-related
**Lp_c25811p401**	367	0.436	0.439	0.005	100	0.040	0.039	-0.020		all				0.023		alto/keto reductase family/NAD(P)-linked oxidoreductase superfamily
**Lp_c26195p495**	367	0.093	0.108	0.141	100	0.010	0.010	-0.005		species/Lp				0.964		iron binding zinc finger CDGSH type/similar to kinesin like protein
**Lp_c26630p901**	356	0.404	0.412	0.016						Lp						peptidase c65 otubain/ubiquitin thioesterase otubain
**Lp_c26844p544**	365	0.334	0.304	-0.102	100	0.000	0.000			species/Lp				0.243		ornithine-delta-aminotransferase/aminotransferase class III
**Lp_c26937p895**	330	0.342	0.369	0.072						Lp						pollen Ole e 1 allergen and extensin family protein
**Lp_c27142p1396**	363	0.416	0.355	-0.174						Lp						ATPase/hydrogen-exporting ATPase/phosphenolpyruvate carboxylase
**Lp_c27765p734**	361	0.524	0.501	-0.047	99	0.515	0.492	-0.051		all				0.731		late embryogenesis abundant protein (LEA) family protein
**Lp_c28145p330**	367	0.319	0.340	0.061	100	0.320	0.297	-0.084	LPL6	all			1	0.022	Positive	NifU-like protein 2
**Lp_c28145p756**	368	0.245	0.258	0.052	100	0.010	0.010	-0.005	LPL6	species/Lp				0.445		NifU-like protein 2
**Lp_c28426p547**	368	0.188	0.175	-0.076	100	0.000	0.000			species/Lp				0.879		ribosomal protein L16p/L10e family
**Lp_c28483p683**	352	0.497	0.479	-0.039						Lp						Protein of unknown function (DUF760)
**Lp_c29024p781**	361	0.504	0.498	-0.013	100	0.300	0.297	-0.016		all				0.65		GDP-mannose 3,5-epimerase/NAD
**Lp_c30198p360**	368	0.380	0.422	0.097	100	0.010	0.010	-0.005		species/Lp				0.324		prohibitin
**Lp_c30954p847**	367	0.433	0.408	-0.064	100	0.000	0.000			species/Lp				0.12		BSD domain-containing protein
**Lp_c31191p1428**	366	0.331	0.359	0.077	100	0.010	0.010	-0.005		species/Lp				0.081		Duplicated homeodomain-like superfamily protein
**Lp_c31206p1180**	368	0.397	0.406	0.022	100	0.110	0.104	-0.058		all				0.002		Protein of unknown function (DUF1423)/potyviral VPg interacting protein 1
**Lp_c31229p1539**	368	0.473	0.461	-0.028	100	0.130	0.122	-0.070		all			1	0.08		galactose oxidase/kelch repeat
**Lp_c31410p562**	360	0.472	0.484	0.023	100	0.010	0.010	-0.005		species/Lp				0.066		aldehyde dehydrogenase family
**Lp_c31430p340**	367	0.417	0.456	0.084	100	0.450	0.494	0.085		all	1			0.313	Negative	coatomer beta subunit
**Lp_c31693p433**	368	0.063	0.061	-0.032	100	0.230	0.220	-0.051		all				0.683		fatty acid desaturase 2 (FAD2)
**Lp_c32359p509**	368	0.519	0.501	-0.038	100	0.020	0.020	-0.010		all				0.707		ribosomal L28 family
**Lp_c32518p1291**	368	0.478	0.495	0.033	100	0.040	0.039	-0.020		all				0.32		glycosyl transferase family 8/plant glycogenin-like starch initiation protein
**Lp_c32989p661**	368	0.342	0.350	0.020	100	0.040	0.039	-0.020		all				0.564		NAD(P)-binding Rossmann-fold/3-beta hydroxysteroid dehydrogenase/isomerase
**Lp_c33326p690**	367	0.409	0.402	-0.018	100	0.370	0.316	-0.179		all	1			0.13	Negative	heavy metal transport/myb-like HTH transcriptional regulator family protein
**Lp_c33380p839**	368	0.481	0.498	0.034	100	0.070	0.086	0.186		all				0.593		Aluminum induced protein with YGL and LRDR motifs
**Lp_c33543p797**	367	0.455	0.495	0.080	100	0.040	0.039	-0.020		all				0.986		similar to Arabisopsis thaliana At1g68490
**Lp_c33997p220**	368	0.280	0.268	-0.047	100	0.500	0.463	-0.085		all				0.353		ATP synthase alpha/beta family
**Lp_c34571p636**	366	0.197	0.191	-0.033	100	0.080	0.077	-0.042		all	1			0.353	Negative	cytochrome B5 isoform B
**Lp_c34658p813**	356	0.503	0.483	-0.043	100	0.010	0.010	-0.005		species/Lp				0.28		triosephosphate isomerase
**Lp_c35003p642**	366	0.350	0.336	-0.043	100	0.000	0.000			species/Lp				0.295		Oxygen evolving enhancer protein 3
**Lp_c35144p736**	352	0.466	0.484	0.036	99	0.374	0.443	0.153		all				0.013		S1/P1 nuclease/endonuclease 4
**Lp_c38820p1995**	353	0.544	0.496	-0.099	99	0.051	0.049	-0.026	LP03	all				0.525		SGNH hydrolase-type esterase superfamily protein
**Lp_c38837p2392**	367	0.259	0.272	0.047	100	0.040	0.039	-0.020		all				0.385		TUDOR-SN protein 1/Staphylococcal nuclease homologue
**Lp_c41343p893**	367	0.504	0.501	-0.008	100	0.290	0.290	-0.004		all				0.048		LUC7 N_terminus domain-containing protein
**Lp_c42715p803**	368	0.043	0.043	-0.022						Lp						importin alpha isoform/armadillo/beta-catenin-like repreat
**Lp_c44052p679**	367	0.515	0.472	-0.093	100	0.050	0.049	-0.026	LP05	all				0.123		UDP-glucuronate decarboxylase/dehydrogenase-isomerase family
**Lp_c46762p868**	365	0.348	0.302	-0.154						Lp						nucleic-acid binding domain OB-fold-like protein
**Lp_c47525p1103**	368	0.443	0.387	-0.145	98	0.000	0.000			species/Lp				0.088		Protein of unknown function (DUF1218)
**Lp_c49110p1018**	366	0.404	0.333	-0.217	99	0.202	0.199	-0.023		all				0.011		ATP synthase/gamma subunit/Cellulose synthase family protein
